# Thermal-Robust Phenoxyimine Titanium Catalysts Bearing Bulky Sidearms for High Temperature Ethylene Homo-/Co- Polymerizations

**DOI:** 10.3390/polym16070902

**Published:** 2024-03-25

**Authors:** Xin Wang, Lishuang Ma, Xiaohua Wang, Wenpeng Zhao, Heng Liu, Xuequan Zhang, Feng Wang

**Affiliations:** 1 Shandong Provincial College Laboratory of Rubber Material and Engineering, Qingdao University of Science & Technology, Qingdao 266042, China; 15615420661@163.com (X.W.); zwp@qust.edu.cn (W.Z.); hengliu@qust.edu.cn (H.L.); xqzhang@qust.edu.cn (X.Z.); 2 Key Laboratory of Rubber-Plastics, Ministry of Education, School of Polymer Science and Engineering, Qingdao University of Science & Technology, Qingdao 266042, China; 3 College of Chemistry and Chemical Engineering, China University of Petroleum (East China), Qingdao 266580, China

**Keywords:** titanium catalyst, phenoxyimine, ethylene polymerization, solution polymerization, thermal robustness

## Abstract

A family of titanium complexes (**Ti1**–**Ti7**) with the general formula LTiCl_3,_ supported by tridentate phenoxyimine [O^−^NO] ligands (**L1**–**L7**) bearing bulky sidearms, were synthesized by treating the corresponding ligands with stoichiometric amount of TiCl_4_. All the ligands and complexes were well characterized by ^1^H and ^13^C NMR spectroscopies, in which *ortho*- methoxyl groups on *N*-aryl moieties shifted to downfield, corroborating the successful coordination reaction. Structural optimization by DFT calculations revealed that one of the phenyl groups on dibenzhydryl moiety could form π-π stacking interaction with the salicylaldimine plane, because of which the obtained titanium complexes revealed good thermal stabilities for high-temperature polymerization of ethylene. The thermal robustness of the complexes was closely related to the strength of π-π stacking interactions, which were mainly influenced by the substituents on the dibenzhydryl moieties; **Ti1**, **Ti4** and **Ti5** emerged as the three best-performing complexes at 110 °C. With the aid of such π-π stacking interactions, the complexes were also found to be active at >150 °C, although decreased activities were witnessed. Besides homopolymerizations, complexes **Ti1**–**Ti7** were also found to be active for the high-temperature copolymerization of ethylene and 1-octene, but with medium incorporation percentage, demonstrating their medium copolymerization capabilities.

## 1. Introduction

The current plastic industry is still dominated by polyolefins, with polyethylene’s (PE’s) production capacity being the highest, reaching ca. 150 million metric tons in 2022. Generally, PE can be industrially produced by a variety of processes, including gas-phase, slurry, solution and high-pressure processes [1]. Among them, the solution process requires the monomers, catalysts, and the obtained polymer products to be soluble in the solvent, which, therefore, demands a high-temperature polymerization condition (≥100 °C). In contrast to other processes, the homogeneous nature of the solution process significantly enhances control over the whole polymerization process, such as heat transfer, mass transfer, etc., and fine-regulating the polymer properties, such as molecular weight, polydispersities, *stereo*-regularities, copolymer compositions, etc. Additionally, the high temperature condition also accompanies other unique advantages: (1) accelerating the heat dissipation because of the increased temperature gradient between the reactor and cooling system; (2) reducing the viscosity of the polymerization medium for better mass transportation; (3) lowering the energy cost for recovering the polymer from the solvent. Because of these features, the solution process is capable of preparing a wide variety of ethylene polymers, ranging from crystalline PE plastics, e.g., high-density polyethylene (HDPE), linear-low-density polyethylene (LDPE), etc., to amorphous ethylene-based elastomers, e.g., ethylene–propylene rubber (EPR), ethylene/*α*-olefin copolymer elastomers (also known as polyolefin elastomer, POE), etc. Nevertheless, the high-temperature condition in the solution process greatly challenges the thermo-stability of the catalytic system because the catalytic activities and molecular weight of the resultant polymers often respond to a sharp decline by increasing the reactor temperature; thermal-robust catalysts capable of producing high-molecular-weight polymers have long been pursued by scientists in this field.

**Chart 1:**
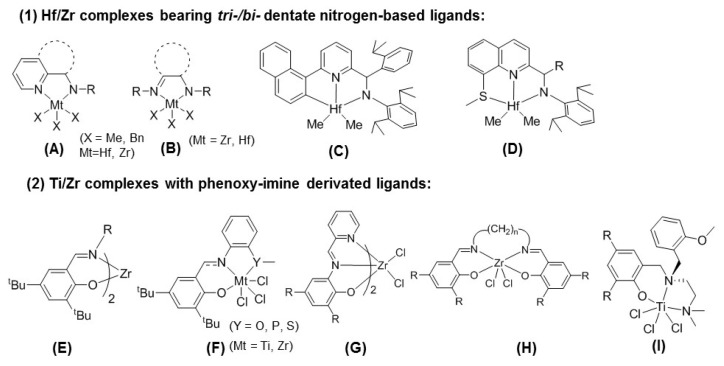
Post-metallocene complexes for high-temperature olefin polymerizations.

So far, metallocene group IV complexes have the workhorse for high-temperature solution polymerization of ethylene [2,3,4,5,6,7,8,9,10,11,12,13]. Some representative catalytic systems are constrained-geometry catalysts (CGC) [14,15,16,17,18], bridged metallocene, half-metallocene [19], that have been developed by Dow, Exxon, Lanxess and other chemical companies or research groups to promote high-temperature ethylene photopolymerization or copolymerization with propylene or *α*-olefins. In contrast, post-metallocene group IV complexes, despite their broader structural diversities, are much less developed. The forefront of these developing postmetallocenes are mainly two types of complexes, i.e., Hf/Zr complexes bearing *tri-/bi-* dentate nitrogen-based ligands (**A**–**D**) [20,21,22,23,24,25,26,27,28,29,30,31,32,33] and Ti/Zr complexes supported by phenoxy-imine-derived ligands (**E**–**I**) [34,35,36,37,38] (Chart 1). Generally, the former type possesses very good *α*-olefin copolymerization abilities, whereas the latter one’s copolymerization ability is very limited; by using such a discrepancy, Dow developed a novel technology called chain-shuttling polymerization via a high-throughput screening strategy to produce olefin block copolymers (OBC) [39,40]. Despite such great advances, most of the phenoxy-imine (amine) Ti/Zr complexes are reported to promote high-temperature olefin polymerization at a range of 90–110 °C, and very few of them can be implemented at ≥130 °C [36]. A breakthrough was recently achieved by Tang et al. in this field; by employing a sidearmed phenoxy-amine structure, the corresponding titanium complexes **F** were able to promote ethylene polymerization at temperatures as high as 150 °C [35]. From the viewpoint of industrialization, a higher temperature is more beneficial for continuous solution polymerizations because it could significantly reduce the viscosity of the polymerization medium for better mass or heat transportation. In this research, we disclose a series of tridentate phenoxyimine [O^−^NO]-based titanium complexes; by introducing a sterically congested dibenzhydryl or dibenzosuberyl moiety, such complexes could promote ethylene homo-/co- polymerizations at a temperature as high as 150 °C, which shows a good strategy for the future design of thermal-robust titanium complexes.

## 2. Materials and Methods

### 2.1. Materials

All manipulations of moisture-/oxygen-sensitive materials were performed with standard Schlenk techniques or in a glovebox. The solvents, including toluene and hexane, were refluxed over sodium–benzophenone and distilled. CDCl_3_ and 1-octene were dried overnight with CaH_2,_ distilled, and degassed by three freeze-pump-thaw cycles prior to use. Methylaluminoxane (MAO, 1.5 M in toluene) was purchased from Akzo Nobel Chemicals Inc. (Amsterdam, The Netherlands) and 1-octene was purchased from Sigma-Aldrich (St. Louis, MO, USA). Other chemicals were purchased from Acros (Beijing, China), Energy Chemical (Shanghai, China) and local suppliers. NMR spectra were recorded on a Bruker AVANCE NEO 400 MHz NMR spectrometer (Karlsruhe, Germany). Fourier transform infrared (FT-IR) spectroscopy was performed using a BRUKE VERTEX-70 spectrophotometer (Karlsruhe, Germany). Mass spectra were recorded by a mass spectrometer with model LCQFleet, samples were dissolved in toluene in the glovebox, direct injection was used, and ionization was performed as an APCI source. Gel permeation chromatography (GPC) was carried out in 1,2,4-trichlorobenzene at 150 °C on the Agilent 1260 infinity II HT GPC instrument (Santa Clara, CA, USA). The data reported were determined via triple detection. Differential scanning calorimetry (DSC) was performed using a TA DSC 25 differential scanning calorimeter (TA, New Castle, DE, USA) that was calibrated using high-purity indium at a heating rate of 10 °C/min. Melting points were determined from the second scan at a heating rate of 10 °C/min following a slow cooling rate of 10 °C/min to remove the influence of thermal history.

### 2.2. Synthesis of the Ligands and Complexes

#### 2.2.1. Synthesis of Ligand **L1**

To a 100 mL round bottom flask, 3,5-di-tert-butyl-2-hydroxybenzaldehyde (2.0 g, 8.5 mmol), 2-benzhydryl-4,6-dimethoxyaniline (2.7 g, 8.5 mmol), *p*-toluenesulfonic acid (0.16 g, 0.85 mmol), and EtOH (100 mL) were added. The reaction was refluxed at 120 °C for 12 h; after the solvent was partially removed, recrystallization from a concentrated ethanol solution gave the target product in a high yield. Yield: 3.5 g, (78.9%). ^1^H NMR (400 MHz, Chloroform-*d*) δ 8.42 (s, 1H, C*H*), 7.37 (d, *J* = 2.4 Hz, 1H, *H*_Ar_), 7.29 (d, *J* = 7.1 Hz, 4H, *H*_Ar_), 7.20 (t, *J* = 7.3 Hz, 2H, *H*_Ar_), 7.12 (d, *J* = 7.1 Hz, 4H, *H*_Ar_), 7.08 (d, *J* = 2.5 Hz, 1H, *H*_Ar_), 6.75 (s, 1H, *H*_Ar_), 6.55 (s, 1H, *H*_Ar_), 5.88 (s, 1H, C*H*), 3.91 (s, 3H, OC*H*_3_), 3.77 (s, 3H, OC*H*_3_), 1.45 (s, 9H, C(C*H*_3_)_3_), 1.30 (s, 9H, C(C*H*_3_)_3_). ^13^C NMR (126 MHz, Chloroform-*d*) δ 161.82, 158.38, 156.65, 152.51, 143.87, 140.07, 136.83, 130.21, 129.42, 128.27, 127.26, 126.33, 126.20, 125.15, 121.58, 118.69, 96.66, 56.28, 56.11, 49.09, 35.14, 34.20, 31.58, 29.53. FT-IR (KBr; cm^−1^): 3481 (m), 2960 (s), 2360 (m), 1617 (m, *v*_C=N_), 1505 (m), 1468 (m), 1437 (m), 1402 (s), 1436 (w), 1300 (m), 1167 (m), 1105 (m), 892 (w), 820 (w), 746 (s), 701 (m), 568 (w). MS (APCI *m*/*z*): Calculated for C_36_H_41_NO_3_ [(M+H])^+^]: 535.32, found 536.32.

#### 2.2.2. Synthesis of Ligand **L2**

The synthesis step is the same as **L1**. Yield: 3.1 g, (70.8%). ^1^H NMR (400 MHz, Chloroform-*d*) δ 8.43 (s, 1H, C*H*), 7.37 (d, *J* = 2.4 Hz, 1H, *H*_Ar_), 7.08 (d, *J* = 7.9 Hz, 5H, *H*_Ar_), 7.00 (d, *J* = 8.0 Hz, 4H, *H*_Ar_), 6.75 (s, 1H, *H*_Ar_), 6.54 (s, 1H, *H*_Ar_), 5.81 (s, 1H, C*H*), 3.90 (s, 3H, OC*H*_3_), 3.77 (s, 3H, OC*H*_3_), 2.32 (s, 6H, C*H*_3_), 1.45 (s, 9H, C(C*H*_3_)_3_), 1.30 (s, 9H, C(C*H*_3_)_3_). ^13^C NMR (126 MHz, Chloroform-*d*) δ 160.74, 157.24, 155.49, 151.22, 139.94, 138.92, 135.70, 134.41, 129.09, 128.12, 127.83, 126.09, 125.19, 124.41, 120.44, 117.56, 95.50, 55.15, 55.01, 47.05, 34.00, 33.07, 30.44, 28.39, 19.96. FT-IR (KBr; cm^−1^): 3488 (m), 2958 (s), 2365 (m), 1614 (m, *v*_C=N_), 1510 (m), 1460 (m), 1289 (m), 1205 (m), 1105 (m), 1030 (m), 895 (w), 817 (w), 563 (w). MS (APCI *m*/*z*): Calculated for C_38_H_45_NO_3_ [(M+H])^+^]: 564.35, found 564.35.

#### 2.2.3. Synthesis of Ligand **L3**

The synthesis step is the same as **L1**. Yield: 3.2 g, (66.8%). ^1^H NMR (400 MHz, Chloroform-*d*) δ 8.32 (s, 1H, C*H*), 7.38 (d, *J* = 2.5 Hz, 1H, *H*_Ar_), 7.33 (s, 2H, *H*_Ar_), 7.17–7.07 (m, 6H, *H*_Ar_), 7.05 (d, *J* = 2.4 Hz, 1H, *H*_Ar_), 6.71 (s, 1H, *H*_Ar_), 6.47 (s, 1H, *H*_Ar_), 5.44 (s, 1H, C*H*)), 3.85 (s, 3H, OC*H*_3_), 3.66 (s, 3H, OC*H*_3_), 3.47–3.37 (m, 2H, C*H*_2_), 2.84–2.75 (m, 2H, C*H*_2_), 1.45 (s, 9H, C(C*H*_3_)_3_), 1.31 (s, 9H, C(C*H*_3_)_3_). ^13^C NMR (126 MHz, Chloroform-*d*) δ 160.81, 157.24, 155.34, 151.19, 139.00, 138.89, 138.85, 135.79, 130.75, 129.20, 128.59, 126.17, 125.76, 125.14, 124.83, 124.66, 120.59, 117.53, 96.06, 55.15, 54.59, 52.80, 34.03, 33.09, 31.15, 30.46, 28.42. FT-IR (KBr; cm^−1^): 2959 (s), 1613 (m, *v*_C=N_), 1607 (m), 1464 (m,), 1440 (m), 1393 (w), 1362 (w), 1302 (m), 1247 (w), 1204 (m), 1165 (m), 1109 (w), 1030 (m), 900 (w), 875 (w), 828 (w), 771 (w), 751 (w), 650 (w), 579 (w). MS (APCI *m*/*z*): Calculated for C_38_H_43_NO_3_ [(M+H])^+^]: 562.33, found 562.33.

#### 2.2.4. Synthesis of Ligand **L4**

The synthesis step is the same as **L1**. Yield: 3.9 g, (79.1%). ^1^H NMR (400 MHz, Chloroform-*d*) δ 13.33 (s, 1H, O*H*), 8.26 (s, 1H, C*H*), 7.39 (d, *J* = 2.5 Hz, 1H, *H*_Ar_), 7.22–7.11 (m, 12H, *H*_Ar_), 7.07–7.02 (m, 4H *H*_Ar_), 7.02–6.96 (m, 4H *H*_Ar_), 6.90 (d, *J* = 2.5 Hz, 1H, *H*_Ar_), 6.63 (d, *J* = 1.8 Hz, 1H, *H*_Ar_), 6.38 (d, *J* = 1.8 Hz, 1H, *H*_Ar_), 5.73 (s, 1H, C*H*), 5.42 (s, 1H, C*H*), 3.64 (s, 3H, OC*H*_3_), 1.45 (s, 9H, C(C*H*_3_)_3_), 1.29 (s, 9H, C(C*H*_3_)_3_). ^13^C NMR (126 MHz, Chloroform-*d*) δ 168.54, 157.23, 149.36, 142.84, 142.13, 139.83, 138.90, 136.92, 135.72, 134.41, 128.48, 128.23, 127.17, 127.05, 126.53, 125.75, 125.21, 125.00, 122.75, 117.05, 110.48, 55.63, 54.85, 50.98, 34.03, 33.07, 30.44, 28.37. FT-IR (KBr; cm^−1^): 2958 (s), 2362 (m), 1619 (m, *v*_C=N_), 1569 (w), 1495 (m), 1452 (m), 1392 (w), 1358 (w), 1254 (m), 1202 (m), 1172 (m), 1140 (m), 1076 (m), 1025 (w), 841 (w), 810 (w), 742 (m), 696 (m), 640 (w). MS (APCI *m*/*z*): Calculated for C_48_H_49_NO_2_ [(M+H])^+^]: 672.38, found 672.38.

#### 2.2.5. Synthesis of Ligand **L5**

The synthesis step is the same as **L1**. Yield: 2.9 g, (65.6%). ^1^H NMR (400 MHz, Chloroform-*d*) δ 13.99 (s, 1H, O*H*), 8.40 (s, 1H, C*H*), 7.38 (d, *J* = 2.5 Hz, 1H, *H*_Ar_), 7.32–7.26 (m, 4H, *H*_Ar_), 7.22 (t, *J* = 7.3 Hz, 2H, *H*_Ar_), 7.11–7.06 (m, 5H, *H*_Ar_), 6.79 (s, 1H, *H*_Ar_), 6.62 (s, 1H, *H*_Ar_), 5.63 (s, 1H, C*H*), 3.87 (s, 3H, OC*H*_3_), 2.24 (s, 3H, CC*H*_3_), 1.45 (s, 9H, C(C*H*_3_)_3_), 1.30 (s, 9H, C(C*H*_3_)_3_). ^13^C NMR (126 MHz, Chloroform-*d*) δ 162.30, 157.42, 149.81, 142.31, 139.00, 135.79, 134.79, 134.18, 133.84, 128.45, 127.33, 126.42, 125.36, 125.30, 120.28, 117.43, 113.07, 54.90, 51.87, 34.02, 33.07, 30.44, 28.40, 18.99. FT-IR (KBr; cm^−1^): 2962 (s), 2363 (m), 1618 (m, *v*_C=N_), 1598 (m), 1503 (m), 1457 (m), 1394 (w), 1364 (w), 1271 (m), 1249 (m), 1197 (w), 1164 (m), 1089 (m), 1026 (m), 974 (w), 881 (w), 843 (m), 744 (w), 701 (m), 644 (w), 570 (m). MS (APCI *m*/*z*): Calculated for C_36_H_41_NO_2_ [(M+H])^+^]: 520.32, found520.32.

#### 2.2.6. Synthesis of Ligand **L6**

The synthesis step is the same as **L1**. Yield: 2.7 g, (62.1%). ^1^H NMR (400 MHz, Chloroform-*d*) δ 14.03 (s, 1H, O*H*), 8.36 (s, 1H, C*H*), 7.29 (d, *J* = 7.0 Hz, 6H, *H*_Ar_), 7.20 (t, *J* = 7.2 Hz, 2H, *H*_Ar_), 7.11 (d, *J* = 7.1 Hz, 3H, *H*_Ar_), 7.06 (s, 1H, *H*_Ar_), 6.90 (s, 1H, *H*_Ar_), 6.72 (s, 1H, *H*_Ar_), 6.55 (s, 1H, *H*_Ar_), 5.87 (s, 1H, C*H*), 3.92 (s, 3H, OC*H*_3_), 3.76 (s, 3H, OC*H*_3_), 2.26 (s, 3H, C*H*_3_), 2.18 (s, 6H, *H*_Ad_), 2.06 (d, *J* = 10.2 Hz, 3H, *H*_Ad_), 1.79 (q, *J* = 12.6 Hz, 6H, *H*_Ad_). ^13^C NMR (126 MHz, Chloroform-*d*) δ 161.49, 158.58, 156.59, 152.41, 143.75, 137.48, 130.74, 130.06, 129.76, 129.32, 128.17, 126.61, 126.11, 125.07, 121.47, 119.09, 96.49, 77.19, 56.19, 56.02, 49.03, 40.25, 37.16, 36.93, 29.10, 20.66. FT-IR (KBr; cm^−1^): 2910 (s), 2361 (s), 1618 (m *v*_C=N_), 1540 (w), 1503 (m), 1455 (m), 1395 (w), 1337 (w), 1287 (m), 1250 (w), 1209 (m), 1167 (w), 1102 (w), 1036 (m), 893 (w), 819 (w), 748 (w), 697 (m), 669 (w), 570 (w). MS (APCI *m*/*z*): Calculated for C_39_H_41_NO_3_ [(M+H])^+^]: 572.32, found 572.32.

#### 2.2.7. Synthesis of Ligand **L7**

The synthesis step is the same as **L1**. Yield: 3.6 g, (71.4%). ^1^H NMR (400 MHz, Chloroform-*d*) δ 14.45 (s, 1H, O*H*), 8.52 (s, 1H, C*H*), 7.64 (d, *J* = 7.5 Hz, 2H, *H*_Ar_), 7.48–7.27 (m, 8H, *H*_Ar_), 7.21 (t, *J* = 7.1 Hz, 3H, *H*_Ar_), 7.11 (d, *J* = 7.4 Hz, 4H, *H*_Ar_), 6.94 (t, *J* = 7.3 Hz, 1H, *H*_Ar_), 6.80 (s, 1H, *H*_Ar_), 6.54 (s, 1H, *H*_Ar_), 5.87 (s, 1H, C*H*), 3.89 (s, 3H, OC*H*_3_), 3.77 (s, 3H, OC*H*_3_). ^13^C NMR (126 MHz, Chloroform-*d*) δ 159.95, 158.64, 157.04, 152.77, 143.68, 137.96, 133.32, 131.13, 129.92, 129.38, 129.32, 128.98, 128.22, 128.09, 127.02, 126.18, 125.14, 121.46, 119.78, 118.48, 96.34, 56.04, 56.01, 49.05. FT-IR (KBr; cm^−1^): 3023 (w), 1607 (m, *v*_C=N_), 1500 (m), 1452 (m), 1430 (m), 1399 (w), 1326 (w), 1281 (m), 1205 (m), 1105 (w), 1075 (w), 1029 (m), 891 (w), 825 (w), 752 (m), 700 (m), 586 (w). MS (APCI *m*/*z*): Calculated for C_34_H_29_NO_3_ [(M+H])^+^]: 500.22, found 500.22.

#### 2.2.8. Synthesis of Ligand **Ti1**

To a solution of ligand **L1** (0.2 g, 0.37 mmol) in 20 mL of toluene, a solution of TiCl_4_ (1.0 mol/L, 0.37 mL, 0.37 mmol) in toluene was added dropwise at 0 °C. Then, the reaction mixture was warmed to room temperature and stirred for 12 h. The solvent was removed under reduced pressure and recrystallization from toluene/n-hexane produced a pure dark red solid. Yield: 0.22 g, (85.6%). ^1^H NMR (500 MHz, Chloroform-*d*) δ 8.11 (s, 1H, *CH*), 7.60 (s, 1H, *H*_Ar_), 7.31 (t, *J* = 7.5 Hz, 4H, *H*_Ar_), 7.25 (s, 2H, *H*_Ar_), 7.21 (s, 1H, *H*_Ar_), 7.09 (d, *J* = 7.7 Hz, 4H, *H*_Ar_), 6.93 (s, 1H, *H*_Ar_), 6.73 (s, 1H, *H*_Ar_), 5.85 (s, 1H, *CH*), 4.83 (s, 3H, *OCH*_3_), 3.81 (s, 3H, *OCH*_3_), 1.53 (s, 9H, *C(CH*_3_*)*_3_), 1.31 (s, 9H, *C(CH*_3_*)*_3_). ^13^C NMR (126 MHz, Chloroform-*d*) δ 160.19, 159.42, 154.98, 152.16, 148.40, 142.46, 136.30, 131.89, 130.22, 129.82, 129.29, 128.51, 127.46, 126.68, 117.67, 96.90, 64.77, 56.43, 49.44, 35.44, 34.77, 31.16, 29.84. FT-IR (KBr; cm^−1^): 2960 (s), 2360 (m), 1613 (m, *v*_C=N_), 1546 (w), 1498 (m), 1464 (m), 1344 (m), 1281 (m), 1249 (m), 1209 (m), 1181 (m), 1014 (w), 875 (m), 759 (m), 700 (m), 584 (w). MS (APCI, *m*/*z*): Calculated for C_36_H_40_Cl_3_NO_3_Ti [(M-2Cl+K)^+^]: 656.18, found 656.73.

#### 2.2.9. Synthesis of Ligand **Ti2**

The synthesis step is the same as **Ti1**. Yield: 0.23 g, (90.6%). ^1^H NMR (500 MHz, Chloroform-*d*) δ 8.13 (s, 1H, *CH*), 7.60 (d, *J* = 2.3 Hz, 1H, *H*_Ar_), 7.23 (d, *J* = 2.3 Hz, 1H, *H*_Ar_), 7.11 (d, *J* = 7.8 Hz, 4H, *H*_Ar_), 6.96 (d, *J* = 7.8 Hz, 4H, *H*_Ar_), 6.93 (s, 1H, *H*_Ar_), 6.70 (s, 1H, *H*_Ar_), 5.78 (s, 1H, *CH*), 4.82 (s, 3H, *OCH*_3_), 3.83 (s, 3H, *OCH*_3_), 2.33 (s, 6H, *CH*_3_), 1.52 (s, 9H, *C(CH*_3_*)*_3_), 1.32 (s, 9H, *C(CH*_3_*)*_3_). ^13^C NMR (126 MHz, Chloroform-*d*) δ 159.16, 158.35, 153.94, 150.98, 147.30, 138.66, 135.28, 135.14, 130.80, 129.18, 128.12, 128.08, 126.43, 125.59, 116.64, 95.71, 63.63, 55.36, 47.48, 34.41, 33.73, 30.11, 28.80, 19.98. FT-IR (KBr; cm^−1^): 2964 (s), 2365 (m), 1610 (m, *v_C=N_*), 1544 (w), 1500 (m), 1464 (m), 1365 (w), 1345 (m), 1281 (m), 1250 (m), 1181 (m), 1094 (w), 1021 (m), 974 (w), 871 (m), 815 (w), 764 (m), 724 (w), 618 (w), 565 (w). MS (APCI, *m*/*z*): Calculated for C_38_H_44_Cl_3_NO_3_Ti [(M-Cl+2H)^+^]: 682.23, found 682.29.

#### 2.2.10. Synthesis of Ligand **Ti3**

The synthesis step is the same as **Ti1**. Yield: 0.24 g, (94.5%).^1^H NMR (400 MHz, Chloroform-*d*) δ 7.99 (s, 1H, *CH*), 7.60 (d, *J* = 2.4 Hz, 1H, *H*_Ar_), 7.32 (d, *J* = 2.2 Hz, 2H, *H*_Ar_), 7.21–7.11 (m, 7H, *H*_Ar_), 6.88 (s, 1H, *H*_Ar_), 6.62 (s, 1H, *H*_Ar_), 5.40 (s, 1H, *CH*), 4.76 (s, 3H, *OCH*_3_), 3.74 (s, 3H, *OCH*_3_), 3.34–3.26 (m, 2H, *CH_2_*), 2.85–2.78 (m, 2H, *CH_2_*), 1.33 (s, 9H, *C(CH*_3_*)*_3_), 1.25 (s, 9H, *C(CH*_3_*)*_3_). ^13^C NMR (126 MHz, Chloroform-*d*) δ 160.26, 159.29, 154.79, 152.05, 148.35, 139.88, 138.64, 136.36, 132.00, 131.89, 130.33, 130.20, 130.04, 129.06, 128.25, 127.37, 127.35, 126.32, 126.01, 117.79, 97.19, 64.71, 55.96, 54.18, 35.45, 34.72, 32.27, 31.14, 29.84. FT-IR (KBr; cm^−1^): 2963 (s), 2358 (m), 1611 (m, *v_C=N_*), 1547 (m), 1488 (m), 1459 (m), 1361 (w), 1331 (w), 1283 (m), 1247 (m), 1201 (m), 1105 (w), 1018 (m), 870 (m), 755 (m), 617 (w), 573 (w). MS (APCI, *m*/*z*): Calculated for C_38_H_42_Cl_3_NO_3_Ti [(M-2Cl)^+^]: 643.23, found 643.28.

#### 2.2.11. Synthesis of Ligand **Ti4**

The synthesis step is the same as **Ti1**. Yield: 0.21 g, (85.7%). ^1^H NMR (400 MHz, Chloroform-*d*) δ 8.14 (s, 1H, C*H*), 7.68 (s, 1H, *H*_Ar_), 7.28–7.20 (m, 13H, *H*_Ar_), 6.94 (s, 8H, *H*_Ar_), 6.79 (s, 1H, *H*_Ar_), 6.68 (s, 1H, *H*_Ar_), 6.57 (s, 1H, *H*_Ar_), 5.44 (d, *J* = 7.9 Hz, 2H, C*H*), 4.64 (s, 3H, OC*H*_3_), 1.54 (s, 9H, C(C*H*_3_)_3_), 1.25 (s, 9H, C(C*H*_3_)_3_). ^13^C NMR (101 MHz, Chloroform-*d*) δ 165.49, 161.16, 152.79, 148.08, 145.43, 142.70, 142.31, 135.96, 135.48, 134.39, 133.03, 131.04, 130.60, 129.37, 129.13, 128.86, 128.58, 127.16, 126.79, 111.30, 64.22, 56.38, 52.54, 35.48, 34.72, 31.11, 29.86. FT-IR (KBr; cm^−1^): 2963 (s), 2359 (m), 1619 (m, *v*_C=N_), 1600 (m), 1548 (m), 1493 (m), 1378 (w), 1366 (w), 1322 (w), 1268(w), 1245 (m), 1181 (m), 1133 (w), 1078 (w), 1044 (m), 913 (w), 860(m), 746 (m), 702 (m), 635 (w), 592 (w). MS (APCI, *m*/*z*): Calculated for C_38_H_48_Cl_3_NO_3_Ti [(M-3Cl)^+^]: 718.32, found 718.15.

#### 2.2.12. Synthesis of Ligand **Ti5**

The synthesis step is the same as **Ti1**. Yield: 0.21 g, (81.4%). ^1^H NMR (400 MHz, Chloroform-*d*) δ 8.14 (s, 1H, C*H*), 7.62 (d, *J* = 2.4 Hz, 1H, *H*_Ar_), 7.33 (t, *J* = 7.4 Hz, 4H, *H*_Ar_), 7.28 (d, *J* = 7.2 Hz, 2H, *H*_Ar_), 7.26 (s, 1H, *H*_Ar_), 7.18 (d, *J* = 2.3 Hz, 1H, *H*_Ar_), 7.09–7.04 (m, 5H, *H*_Ar_), 6.85 (s, 1H, *H*_Ar_), 5.64 (s, 1H, C*H*), 4.79 (s, 3H, OC*H*_3_), 2.28 (s, 3H, C*H*_3_), 1.52 (s, 9H, C(C*H*_3_)_3_), 1.31 (s, 9H, C(C*H*_3_)_3_).^13^C NMR (126 MHz, Chloroform-*d*) δ 160.51, 156.58, 150.64, 148.33, 142.09, 141.16, 139.58, 136.43, 132.41, 131.62, 130.45, 129.47, 128.74, 127.16, 126.93, 117.04, 115.45, 64.56, 53.22, 35.45, 34.74, 31.13, 29.83, 20.48. FT-IR (KBr; cm^−1^): 2960 (s), 2360 (w), 1611 (m, *v_C=N_*), 1549 (m), 1496 (m), 1455 (m), 1364 (m), 1325 (w), 1274 (m). 1245 (m), 1212 (m), 1187 (m), 1072 (w), 1026 (w), 979 (m), 919 (w), 873 (m), 756 (m), 699 (m), 601 (m), 569 (m). MS (APCI, *m*/*z*): Calculated for C_36_H_40_Cl_3_NO_3_Ti [(M)^+^]: 671.16, found 671.02.

#### 2.2.13. Synthesis of Ligand **Ti6**

The synthesis step is the same as **Ti1**. Yield: 0.23 g, (90.6%). ^1^H NMR (500 MHz, Chloroform-*d*) δ 8.03 (s, 1H, C*H)*, 7.31 (t, *J* = 7.2 Hz, 5H, *H*_Ar_), 7.26 (d, *J* = 3.6 Hz, 3H, *H*_Ar_), 7.06 (d, *J* = 6.8 Hz, 4H, *H*_Ar_), 7.02 (s, 1H, *H*_Ar_), 6.87 (s, 1H, *H*_Ar,_), 6.71 (s, 1H, *H*_Ar_), 5.83 (s, 1H, C*H*), 4.82 (s, 3H, OC*H*_3_), 3.82 (s, 3H, OC*H*_3_), 2.34 (s, 3H, C*H*_3_), 2.19 (s, 6H, *H*_Ad_), 1.89 (s, 3H, *H*_Ad_), 1.80 (s, 3H, *H*_Ad_). 1.25 (s, 3H, *H*_Ad_), ^13^C NMR (126 MHz, Chloroform-*d*) δ 160.46, 159.41, 154.50, 152.15, 142.41, 137.36, 135.52, 135.40, 133.21, 129.85, 129.26, 128.51, 127.84, 126.68, 126.54, 117.61, 96.84, 64.65, 56.40, 49.54, 40.86, 37.50, 36.77, 28.88, 20.96. FT-IR (KBr; cm^−1^): 2907 (s), 2360 (w), 1608 (m, *v*_C=N_), 1546 (w), 1490 (m), 1448 (m), 1374 (w), 1333 (m), 1289 (m), 1203 (m), 1099 (w), 1017 (w), 974 (w), 886 (m), 861 (m), 752 (m), 700 (m), 668 (m), 610 (w), 582 (w). MS (APCI, *m*/*z*): Calculated for C_38_H_44_Cl_3_NO_3_Ti [(M-Cl)^+^]: 688.19, found 688.68.

#### 2.2.14. Synthesis of Ligand **Ti7**

The synthesis step is the same as **Ti1**. Yield: 0.23 g, (88.1%). ^1^H NMR (500 MHz, Chloroform-*d*) δ 8.14 (s, 1H, C*H*), 7.69 (dd, *J* = 15.0, 7.6 Hz, 3H, *H*_Ar_), 7.50 (t, *J* = 7.6 Hz, 2H, *H*_Ar_), 7.40 (dd, *J* = 11.5, 7.7 Hz, 2H, *H*_Ar_), 7.32 (t, *J* = 7.6 Hz, 4H, *H*_Ar_), 7.27 (s, 1H, *H*_Ar_), 7.22 (d, *J* = 7.7 Hz, 3H, *H*_Ar_), 7.08 (d, *J* = 7.5 Hz, 4H, *H*_Ar_), 6.92 (s, 1H, *H*_Ar_), 6.72 (s, 1H, *H*_Ar_), 5.84 (s, 1H, C*H*), 4.82 (s, 3H, OC*H*_3_), 3.82 (s, 3H, OC*H*_3_). ^13^C NMR (126 MHz, Chloroform-d) δ 159.59, 154.11, 152.24, 142.29, 137.38, 135.31, 134.34, 129.94, 129.31, 129.20, 128.56, 128.47, 127.96, 127.16, 126.65, 126.31, 125.65, 117.69, 96.84, 64.99, 56.36, 49.54. FT-IR (KBr; cm^−1^): 3024 (m), 1612 (m, *v_C=N_*), 1559 (w), 1499 (m), 1454 (m), 1378 (w), 1341 (w), 1294 (m), 1210 (m), 1093 (w), 1021 (m), 984 (w), 878 (m), 762 (m), 700 (m), 649 (w), 583 (w). MS (APCI, *m*/*z*): Calculated for C_34_H_28_Cl_3_NO_3_Ti [(M)^+^]: 651.06, found 651.31.

### 2.3. DFT Calculation Details

Density functional theory (DFT) calculations were performed to gain deep understanding of the nature of π-π stacking interaction. The geometry structures of all species were obtained by full optimization using the B3LYP functional, including empirical dispersion correction computed with Grimme’s D3 formula (B3LYP-D3) [41]. The all-electron 6–31G(d,p) basis set was applied for H, C and N atoms, while the energy-consistent, fully relativistic DF-adjusted 10-electron-core pseudopotential ECP10MDF with (8s7p6d1f)/[6s5p3d1f] basis sets were employed for Ti, respectively [42]. The solvation effect of toluene was taken into account using the SMD solvation model [43]. All the DFT calculations were performed with the Gaussian 16 program [44]. The reduced density gradient (RDG) analyses was carried out through the Multiwfn 3.8 [45].

## 3. Results and Discussion

### 3.1. Synthesis and Characterization of the Titanium Complexes

**Scheme 1:**
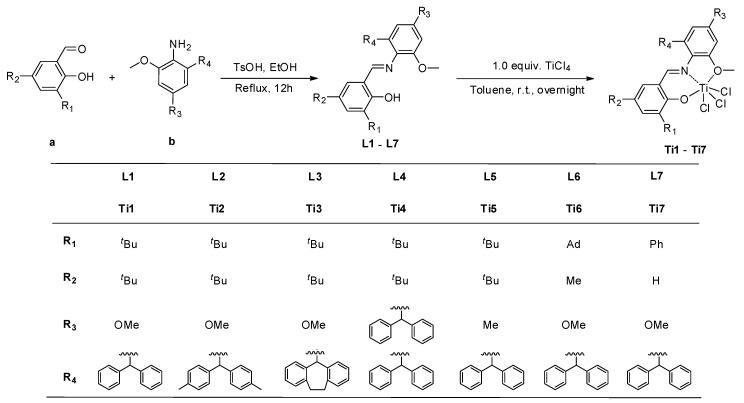
Synthetic procedure for phenoxyimine [O^−^NO] ligands **L1**–**L7** and corresponding titanium complexes **Ti1**–**Ti7.**

**Figure 1:**
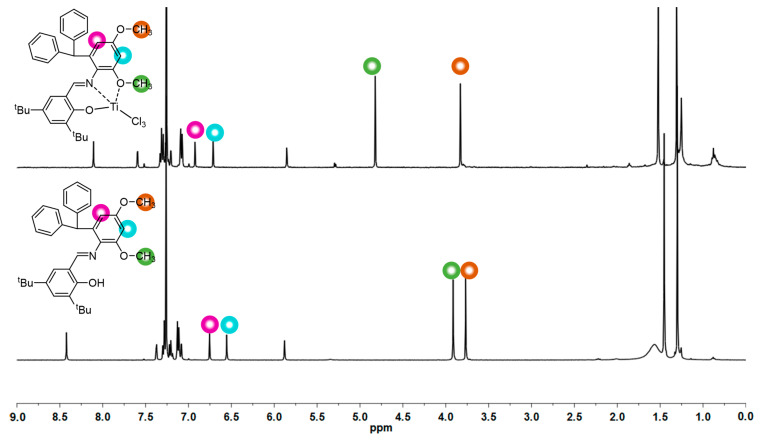
^1^H NMR spectra of ligand **L1** and complex **Ti1** (in CDCl_3_).

**Figure 2:**
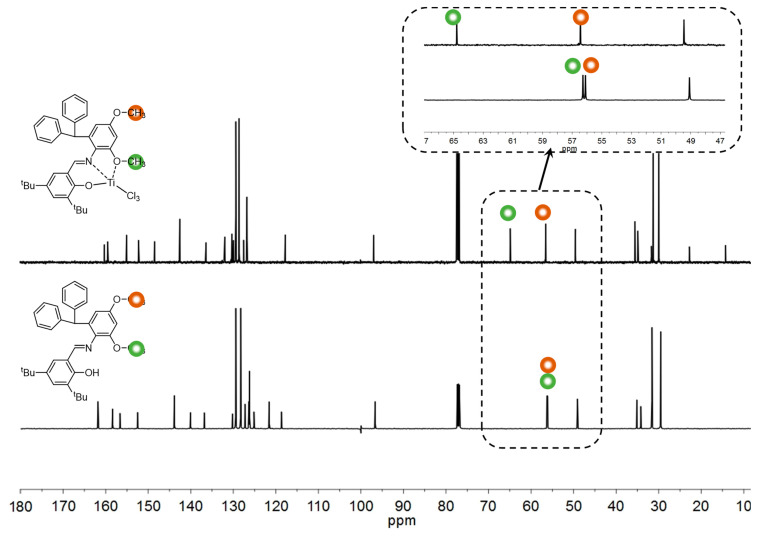
^13^C NMR spectra of ligand **L1** and complex **Ti1** (in CDCl_3_).

The general synthetic route for the targeted titanium complexes **Ti1**–**Ti7** is depicted in Scheme 1. It starts from the bulky amine precursor **b** that is prepared from a Friedel–Crafts reaction between 2-methoxyaniline derivatives and substituted benzhydrols. The subsequent Schiff base condensation reaction between compound **b** and substituted salicylaldehyde compounds **a** results in the formation of the targeted ligands **L1**–**L7** with high yields. All the ligands are well characterized by ^1^H and ^13^C NMR analysis. Directly treating ligands **L1**–**L7** with one equivalent of TiCl_4_ in toluene solution gives rise to the desired complexes **Ti1**–**Ti7** in very high yields. Attempts to characterize their solid-state structures by single-crystal X-ray diffractions failed. Nevertheless, successful complexation between the ligands and TiCl_4_ can also be verified by ^1^H NMR analysis. Figure 1 shows the ^1^H NMR spectra differences between the ligand **L1** and its corresponding complex **Ti1**, from which it can be observed that, after complexation, resonance peaks for *ortho*-and *para*- methoxyl groups shift to a downfield (from 3.91 and 3.77 ppm to 4.83 and 3.81 ppm), hinting at the reduced electron densities therein, caused by the strong *electro*-withdrawing property of the metal center after coordination. Because of the same reason, aryl protons adjacent to methoxyl groups also shift to downfield from 6.75 and 6.55 ppm to 6.93 and 6.72 ppm. ^13^C NMR and FTIR spectra also provide direct evidence of successful complexation. In the ^13^C NMR spectrum, the two methoxyl groups on the *para*- and *ortho*-positions of the ligand **L1** were found to be located at 56.28 and 56.11 ppm, showing striking contrast to their counterparts in the complex **Ti1** that shift to much lower fields of 56.43 and 67.77 ppm, respectively (Figure 2). Especially for the *ortho*-methoxyl, a difference of 11.66 ppm was witnessed. For the FTIR spectrum, the stretching vibration peaks of the imine -C=N group witness a red-shift from 1617 cm^−1^ to 1613 cm^−1^, indicating the reduced electron-density therein, caused by complexation. Other complexes also reveal similar changes.

**Figure 3:**
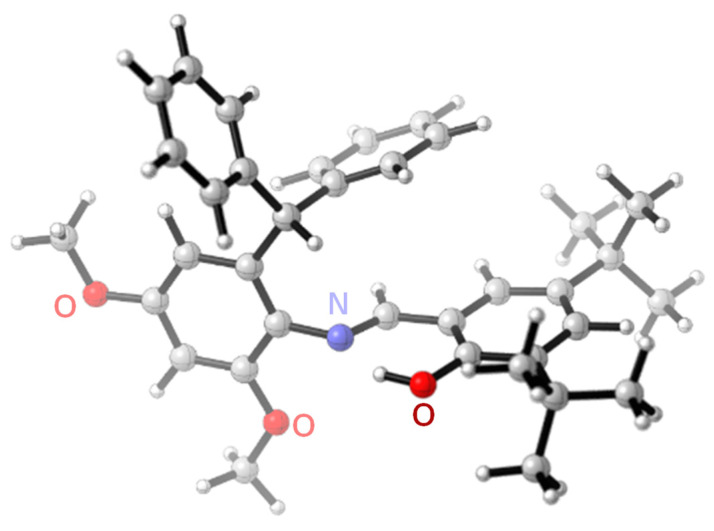
Optimized structure of ligand d **L1** by DFT calculations.

**Figure 4:**
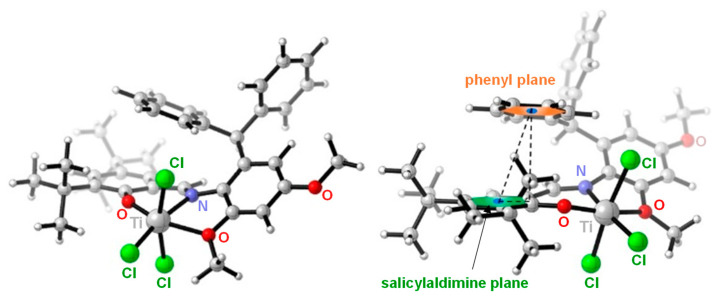
Optimized structure of complex **Ti1** by DFT calculations (**left**: front view; **right**: side view).

**Figure 5:**
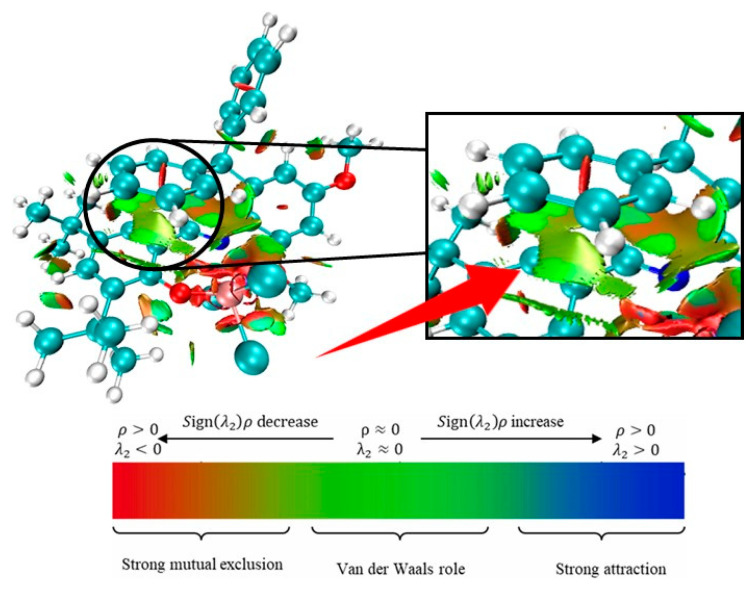
Visualization of intramolecular interaction of complex **Ti1**.

**Figure 6:**
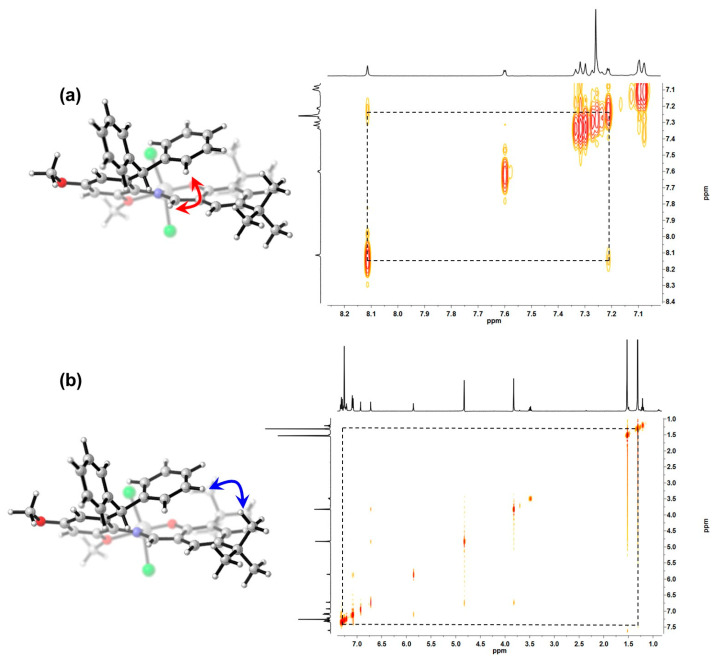
2D NOE NMR for complex **Ti1** ((**a**), red arrow represent a spatial correlation between the imine proton and phenyl protons from the Ph_2_CH- group; (**b**), blue arrow represent a spatial correlation between *tert*-butyl proton and phenyl protons from the Ph_2_CH- group).

**Figure 7:**
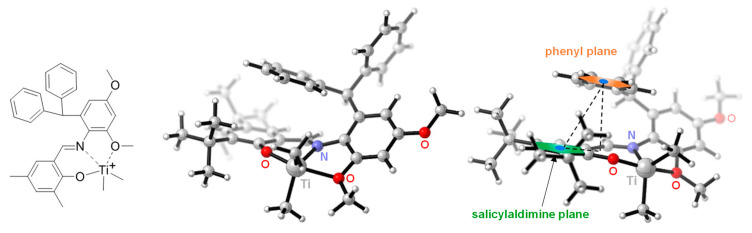
The structure of **Ti1**’s active species (**left**); optimized structure of **Ti1**’s active species by DFT calculations (**middle**: front view; **right**: side view).

**Figure 8:**
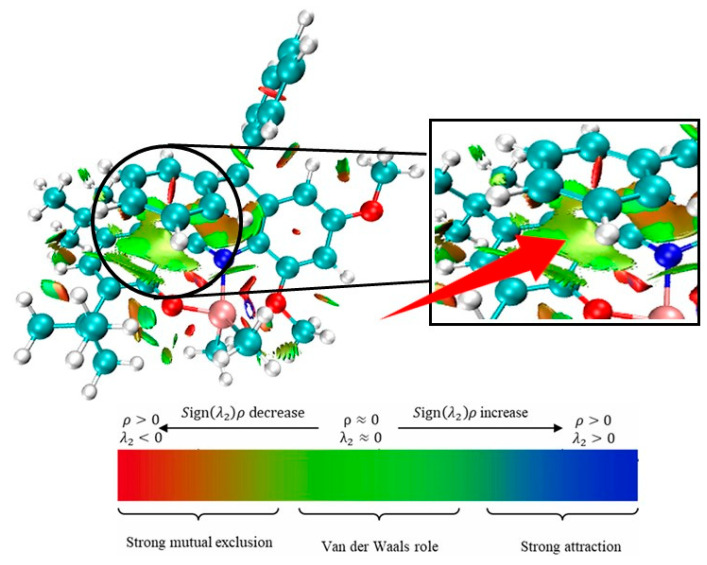
Visualization of intramolecular interaction of **Ti1**’s active species.

The structural optimization of ligand **L1** and complex **Ti1** by DFT calculations was further carried out to acquire more insights into their structural differences after coordination, especially the micro-environment differences around the metal center. As the structure of ligand **L1** shows in Figure 3, *E*- configuration was adopted for the –C=N– imine moiety, forming an intermolecular hydrogen bond between the phenol proton and nitrogen atom from the imine moiety. Moreover, it was found that the *N*-aryl moiety was almost perpendicular to the salicylaldimine plane, giving a dihedral angle of 79.1°, which was similar to many previous reports [46,47,48]^.^ For complex **Ti1**, *E*- configuration was also adopted for the –C=N– imine moiety. Nevertheless, the *ortho*-methoxyl on *N*-aryl rotated towards the salicylaldimine plane to form a sidearm coordination bond with the titanium center. The resultant coordination geometry at the titanium center could be best described as distorted octahedron, with two oppositely orientated chloride atoms forming the two axial points, and with four atoms forming the equatorial plane, including an oxygen atom from phenol, a nitrogen atom from imine, another oxygen atom from *ortho*-methoxyl on *N*-aryl and one chloride atom. Such a geometry was much similar to previous reports [38]. Additionally, for the formed coordination structure in **Ti1**, bond distances of O_(Ph)_–Ti, N_(C=N)_–Ti, and O_(MeO)_–Ti were 1.787 Å, 2.159 Å, 2.199 Å, respectively, which were also close to previously reported values [38]. One prominent difference of **Ti1** to such previously reported analogues was that the *N*-aryl plane was not coplanar with the salicylaldimine plane, giving a dihedral angle of 42.4°. Such a dihedral angle also showed clear contrast to ligand **L1**, wherein the *N*-aryl was nearly perpendicular to the salicylaldimine plane. The reason caused such differences was mainly because of the large size of dibenzhydryl moiety, which showed obvious steric repulsion to adjacent parts of the ligand. One prominent feature that has been observed in **Ti1** is that the phenyl group on the dibenzhydryl moiety can form π-π stacking interaction with the salicylaldimine plane. As shown in Figure 4 (right), the dihedral angle between the phenyl group and the salicylaldimine plane is 17.4°, and the distance from the centroid of the phenyl ring to the salicylaldimine plane is 3.40 Å, which are very similar to π-π stacking characteristics in previous reports [49]. Such a weak intramolecular interaction can be further confirmed by RDG analysis. As shown in Figure 5, the negative regions correspond to areas of electron density concentration associated with attractive interactions; positive regions correspond to areas where electron density is repelling, suggesting steric repulsion or unfavorable interactions. Obvious attractive π-π stacking interaction can be identified between the phenyl moiety and the salicylaldimine plane. Such a π-π stacking interaction can be visualized from 2D NOE NMR of complex **Ti1,** in which a correlation between the imine proton (-C*H*=N-) and phenyl protons from the Ph_2_CH- group can be clearly observed (Figure 6a, red arrow). Correlation between *tert*-butyl proton (*t*Bu) and phenyl protons from the Ph_2_CH- group could also be observed, but showed much weaker interactions (Figure 6b, blue arrow). For **Ti1**’s cationic active species, its structure was also optimized by DFT calculations in order to emphasize the presence of π-π stacking interaction. As the structure shows in Figure 7, the distance from the centroid of the phenyl ring of the dibenzhydryl moiety to the salicylaldimine plane became much smaller, giving a value of 3.23 Å; this indicates that the π-π stacking interaction became much stronger than that in neutral **Ti1**. RDG analysis showed similar results, in which the π-π stacking interaction area became much bigger (Figure 8), also suggesting strengthened interactions.

### 3.2. High-Temperature Ethylene Polymerizations

**Figure 9:**
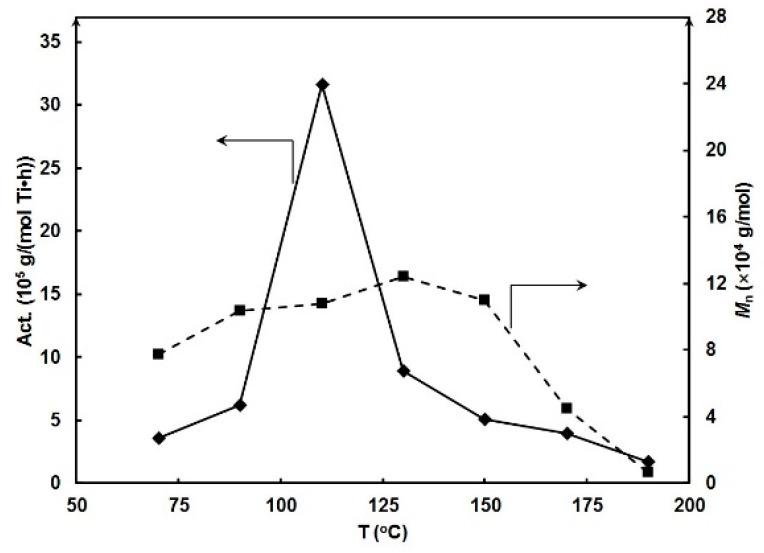
Polymerization activities (**◆**) and *M*_n_s (**■**) of the obtained PEs under different temperatures (solid line represents activity; dot line represent molecular weight).

Ethylene polymerizations using the synthesized titanium complexes **Ti1**–**Ti7** as precatalysts in the presence of methylaluminoxane (MAO) were subsequently evaluated under different conditions, and the results were summarized in Table 1 and Table 2. High-temperature polymerization is a key target in this research; therefore, optimization studies were commenced by carrying out ethylene homopolymerization under different temperatures by using **Ti1** as a catalyst. It was found that, in a ratio of MAO/**Ti1** = 1000, **Ti1** revealed good catalytic activities in a temperature range of 70~190 °C. Increasing the temperature resulted first in the increment and then decrement of the catalytic activities, giving 110 °C as the optimized temperature, which demonstrated a catalytic activity of 31.60 × 10^5^ g·mol_Ti_^−1^·h^−1^ (Figure 9). It is worth mentioning that **Ti1** can successfully survive at extremely high temperatures of 170 and 190 °C, under which most of the titanium catalysts completely deactivate; although, significantly decreased activities (3.92 × 10^5^ and 1.76 × 10^5^ g·mol_Ti_^−1^·h^−1^) were observed. Moreover, the molecular weights of the obtained PEs were also significantly influenced by polymerization temperatures. As shown in Figure 9, PEs’ molecular weight first increased from 7.74 × 10^4^ g/mol (70 °C) to 10.33 × 10^4^ g/mol (90 °C), accompanied by the increased activities; then, slightly changed values in the range of 10.33~12.44 × 10^4^ g/mol were observed, implying that the ratios of chain propagation and chain termination were almost similar in such a range. Nevertheless, when further elevating the temperature to 170 and 190 °C, perhaps due to the sharply facilitated chain terminations or transfers, PEs’ molecular weights witnessed sharp decrements to 4.48 × 10^4^ and 0.63 × 10^4^ g/mol, respectively.

After acquiring the optimized temperature, other polymerization conditions, including MAO/Ti ratios, ethylene pressures, were also carried out. As the data summarized in Table 1 show, increasing the MAO/**Ti1** ratio from 500 to 1000 resulted in monotonous increments of the catalytic activities, giving MAO/**Ti1** = 1000 as the best parameter. Regarding ethylene pressure, increasing it from 5 to 20 atm resulted in gradually enhanced activities, as expected, due to the gradually facilitated chain propagation rates. Because of the same reason, *M*_n_s of the obtained PEs also increased from 3.19 × 10^4^ g/mol to 10.75 × 10^4^ g/mol.

**Figure 10:**
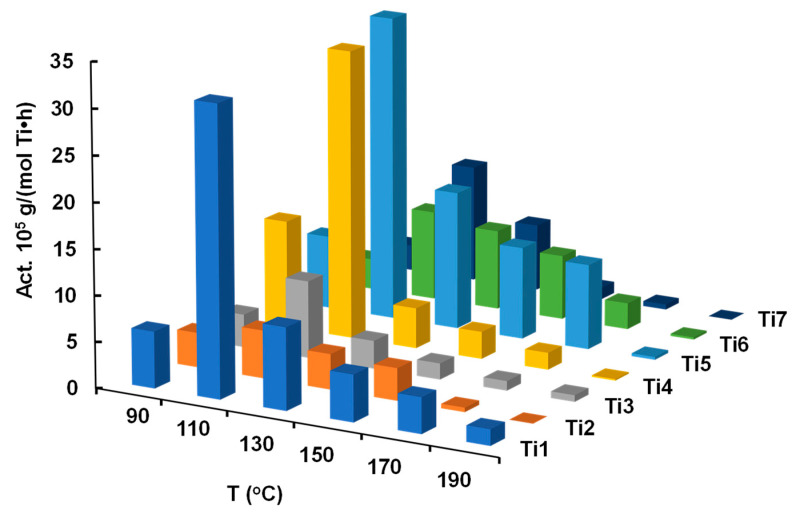
Influence of temperature on the catalytic activities of complexes **Ti1**–**Ti7.**

**Figure 11:**
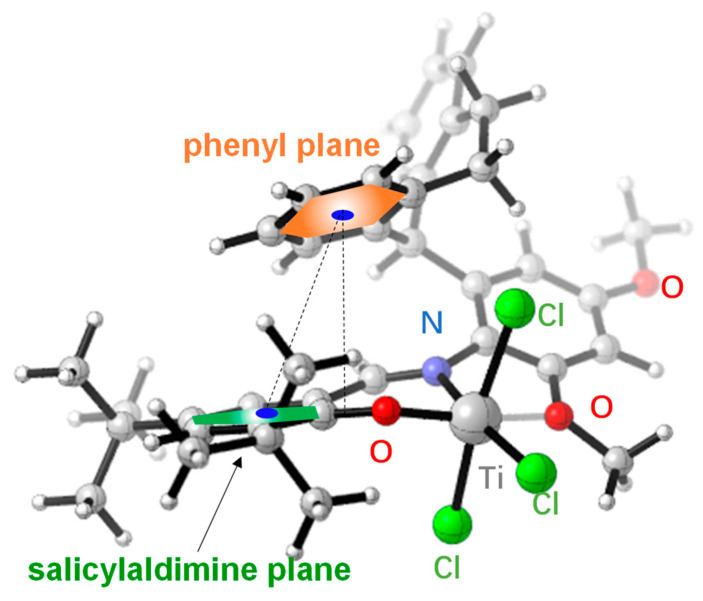
Optimized structure of complex **Ti3** by DFT calculations.

**Figure 12:**
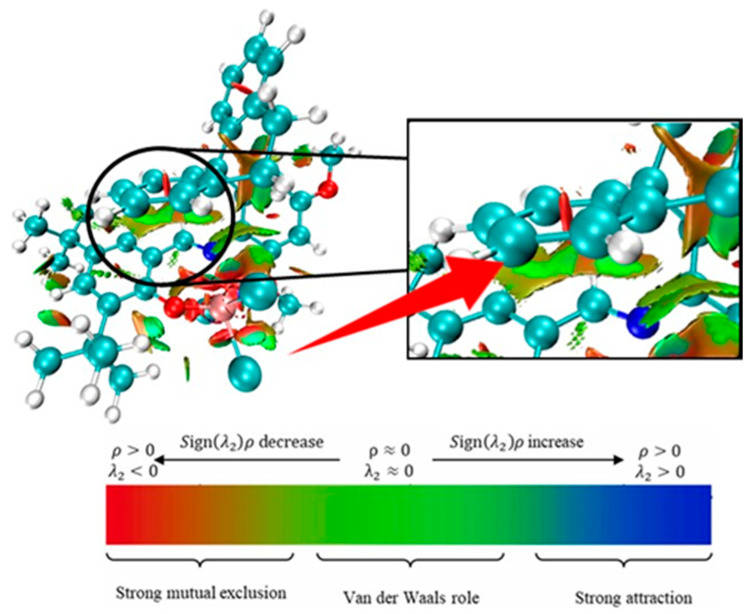
Visualization of intramolecular interaction of complex **Ti3**.

Under the optimal conditions achieved above, high-temperature ethylene polymerization behaviors for all the titanium complexes **Ti1**–**Ti7** were systematically compared in the following studies. All the complexes were evaluated in a temperature range of 90~150 °C. As per the results shown in Table 2 and Figure 10, all the complexes revealed excellent thermal stabilities and subsequently could catalyze ethylene homopolymerization at such high temperatures, giving PE products in mediate-to-high catalytic activities. Additionally, the complexes showed similar trends against temperature variations, i.e., the best catalytic activities were observed at 110 °C; both elevating and lowering the temperature would bring negative influences on the overall catalytic activities (Figure 10). Compared to other sidearmed phenoxyimine titanium complexes that generally demonstrated relatively poorer thermal stabilities, [50] specific reasons that caused such good thermal stabilities for the present complexes **Ti1**–**Ti7** might be related to the π-π stacking interactions formed between the dibenzhydryl moiety and salicylaldimine plane (*vide supra*). Such an intramolecular π-π stacking interaction was able to prohibit the rotation of *N*-aryls and, therefore, inhibit the dissociation of the coordination bond formed between the titanium center and the *ortho*-methoxyl group. Among all the seven titanium complexes, **Ti1**, **Ti4** and **Ti5** were the three best-performing complexes, which showed catalytic activities of 31.60 × 10^5^, 31.52 × 10^5^ and 33.28 × 10^5^ g·mol_Ti_^−1^·h^−1^, respectively; this was probably because the unsubstituted phenyl group on the dibenzhydryl moiety could form effective π-π stacking interaction with the salicylaldimine plane. In contrast, **Ti2** and **Ti3** showed much poorer reactivity than the other five complexes, which only exhibited activities of 5.20 and 8.40 × 10^5^ g·mol_Ti_^−1^·h^−1^, respectively. These inferior results probably originated from the ineffective formation of π-π stacking interactions because of the interference caused by substituents on the phenyl groups. Such speculation can be confirmed by the DFT-optimized structure of **Ti3;** the π-π stacking interaction was much weaker than that in **Ti1**, as revealed from the bigger dihedral angle between phenyl on the dibenzhydryl moiety and salicylaldimine plane (27.9° vs. 17.4°), as well as the longer distance of the centroid of phenyl to salicylaldimine plane (3.62 Å vs. 3.40 Å) (Figure 11). RDG analysis of **Ti3** also showed similar indications (Figure 12).

The present titanium complexes **Ti1**–**Ti7** were also evaluated for high-temperature copolymerizations of ethylene with 1-octene. As the results summarized in Table 3 show, all of them worked well for the copolymerization reactions, giving catalytic activities in a range of 3.20~12.80 × 10^5^ g·mol_Ti_^−1^·h^−1^. For complexes **Ti1**, **Ti4** and **Ti5**, which had revealed high activities during ethylene homopolymerization, they also demonstrated quite high activities in copolymerization, again suggesting the key role of π-π stacking interaction in enhancing the overall thermostabilities of the active species. Nevertheless, compared to homopolymerization, obvious decrements in the activities were also observed in these copolymerization reactions. For instance, for **Ti1**, activity dropped from 31.60 × 10^5^ g·mol_Ti_^−1^·h^−1^ to 10.08 × 10^5^ g·mol_Ti_^−1^·h^−1^ after the incorporation of 0.254 mol/L 1-octene, and for **Ti4**, activity decreased from 31.52 × 10^5^ g·mol_Ti_^−1^·h^−1^ to 12.6 × 10^5^ g·mol_Ti_^−1^·h^−1^; these results suggest the absence of a comonomer effect [51], also hinting at their relatively poorer copolymerization abilities. Such a conclusion could also be drawn from the comonomer incorporation ratios that were calculated from ^1^H NMR, which showed values of 4.1~5.4%; these are generally lower than those of other early transition metal complexes under similar conditions (Supplementary Materials).

## 4. Conclusions

In summary, several titanium (IV) complexes based on tridentate phenoxyimine [O^−^NO] ligands bearing bulky sidearms were synthesized and thoroughly characterized. By introducing such bulky sidearm, π-π stacking interaction can be formed between the phenyl group on dibenzhydryl moiety and the salicylaldimine plane, as revealed by the DFT-optimized structures and RDG analysis. In the presence of MAO, these titanium complexes were able to catalyze ethylene homopolymerizations and copolymerization with 1-octene at high temperatures of 70~190 °C. The optimized temperature was found to be 110 °C, under which complexes **Ti1**, **Ti4** and **Ti5** performed the best catalytic activities due to their relatively stronger π-π stacking interactions, which showed polymerization activity values of 31.60 × 10^5^, 31.52 × 10^5^ and 33.28 × 10^5^ g·mol Ti^−1^·h^−1^, respectively. The complexes also worked for higher temperatures of >110 °C, but exhibited gradually decreased activities. During copolymerization studies, the complexes revealed activities in a range of 3.20~12.80 × 10^5^ g·mol Ti^−1^·h^−1^, but with medium copolymerization capabilities.

## Figures and Tables

**Table 1:** Ethylene polymerization by **Ti1** under different conditions *^a^*.

Entry	T (°C)	Press. (atm)	MAO/Ti	Yield (g)	Act. *^b^*	*M*_n_ *^c^*	*M*_w_ *^c^*	PDI *^c^*	*T*_m_ (°C) *^d^*
1	70	20	1000	0.045	3.60	7.74	46.79	2.86	140.0
2	90	20	1000	0.077	6.16	10.33	46.39	4.49	140.9
3	110	20	1000	0.395	31.60	10.75	47.52	4.42	140.0
4	130	20	1000	0.112	8.96	12.44	48.55	3.90	139.5
5	150	20	1000	0.064	5.12	11.02	39.83	3.62	136.6
6	170	20	1000	0.049	3.92	4.48	42.55	4.58	133.0
7	190	20	1000	0.022	1.76	0.63	0.73	1.17	132.6
8	110	20	500	0.005	0.40	-	-	-	130.6
9	110	20	800	0.084	6.72	6.75	19.04	2.82	136.0
10	110	15	1000	0.180	14.4	11.96	48.15	4.03	136.0
11	110	10	1000	0.032	2.56	4.14	11.74	2.84	134.5
12	110	8	1000	0.029	2.32	5.57	16.52	2.96	141.4
13	110	5	1000	0.019	1.52	3.19	11.66	3.65	137.9

*^a^* Polymerization conditions: 1.5 μmol Ti complex, 50 mL toluene, 5 min. *^b^* Activity (Act.) = 10^5^ g·mol_Ti_^−1^·h^−1^. *^c^ M*_n_: 10^4^ g/mol, *M*_w_ and *M*_w_/*M*_n_ were determined by GPC in trichlorobenzene at 150 °C using polystyrene standards. *^d^ T*_m_ was determined by differential scanning calorimetry (DSC).

**Table 2:** Ethylene polymerization by **Ti1**–**Ti7** under different temperatures *^a^*.

Entry	Cat.	T (°C)	MAO/Ti	Yield (g)	Act. *^b^*	*M*_n_ *^c^*	*M*_w_ *^c^*	PDI *^c^*	*T*_m_ (°C) *^d^*
1	**Ti1**	90	1000	0.077	6.16	10.33	10.33	4.49	140.9
2		110	1000	0.395	31.60	10.75	10.75	4.42	140.0
3		130	1000	0.112	8.96	12.44	12.44	3.90	139.5
4		150	1000	0.064	5.12	11.02	11.02	3.62	136.6
5	**Ti2**	90	1000	0.047	3.76	17.94	17.94	3.94	136.4
6		110	1000	0.065	5.20	10.60	46.12	4.35	136.1
7		130	1000	0.048	3.84	8.56	37.00	4.32	132.6
8		150	1000	0.044	3.52	3.01	26.18	8.47	132.1
9	**Ti3**	90	1000	0.045	3.60	11.48	30.41	2.65	138.7
10		110	1000	0.105	8.40	10.20	35.02	3.44	138.6
11		130	1000	0.038	3.04	12.39	40.21	3.25	138.1
12		150	1000	0.022	1.76	5.62	46.15	8.21	138.8
13	**Ti4**	90	1000	0.147	11.76	9.24	18.16	1.97	141.1
14		110	1000	0.394	31.52	6.16	16.05	2.60	140.1
15		130	1000	0.055	4.40	6.78	34.21	5.05	138.5
16		150	1000	0.037	2.96	7.29	34.20	4.69	137.6
17	**Ti5**	90	1000	0.100	8.00	10.12	34.46	3.41	139.9
18		110	1000	0.416	33.28	21.59	60.03	2.78	139.7
19		130	1000	0.188	15.04	14.54	45.41	3.12	138.7
20		150	1000	0.130	10.04	15.38	47.74	3.11	138.1
21	**Ti6**	90	1000	0.042	3.36	1.01	1.26	1.12	138.7
22		110	1000	0.122	9.76	8.53	37.76	4.42	138.7
23		130	1000	0.109	8.72	6.69	28.09	4.25	138.1
24		150	1000	0.088	7.04	5.17	27.32	5.28	137.9
25	**Ti7**	90	1000	0.035	2.80	8.96	28.31	3.16	139.4
26		110	1000	0.160	12.8	15.87	39.04	2.46	138.8
27		130	1000	0.091	7.28	8.74	32.92	3.77	138.1
28		150	1000	0.016	1.28	1.81	16.59	9.15	138.0

*^a^* Polymerization conditions: 1.5 μmol Ti complex, 50 mL toluene, 5 min. *^b^* Activity (Act.) = 10^5^ g·mol_Ti_^−1^·h^−1^. *^c^ M*_n_: 10^4^ g/mol, *M*_w_ and *M*_w_/*M*_n_ were determined by GPC in trichlorobenzene at 150 °C using polystyrene standards. *^d^ T*_m_ was determined by differential scanning calorimetry (DSC).

**Table 3:** Copolymerization of ethylene with 1-octene by **Ti1**–**Ti7** at high temperatures. *^a^*

Entry	Cat.	1-Oct. (mol/L)	Yield (g)	Act. *^b^*	*M*_n_ *^c^*	*M*_w_ *^c^*	PDI *^c^*	*T*_m_ (°C) *^d^*	χ (%) *^e^*
1	**Ti1**	0.254	0.126	10.08	6.61	17.68	2.67	131.4	4.1
2	**Ti2**	0.254	0.160	12.80	6.45	18.26	2.83	129.9	4.9
3	**Ti3**	0.254	0.040	3.20	4.99	16.21	3.25	127.9	5.3
4	**Ti4**	0.254	0.158	12.6	5.91	18.68	3.16	130.5	4.5
5	**Ti5**	0.254	0.118	9.44	11.79	65.68	5.57	130.5	4.5
6	**Ti6**	0.254	0.134	10.72	6.20	17.20	2.77	133.9	4.0
7	**Ti7**	0.254	0.040	3.20	17.07	96.30	5.64	128.9	5.4

*^a^* Polymerization conditions: 1.5 μmol Ti complex, 50 mL toluene, 5 min, 110 °C. *^b^* Activity (Act.) = 10^5^ g·mol_Ti_^−1^·h^−1^. *^c^ M*_n_: 10^4^ g/mol, *M*_w_ and *M*_w_/*M*_n_ were determined by GPC in trichlorobenzene at 150 °C using polystyrene standards. *^d^ T*_m_ was determined by differential scanning calorimetry (DSC). *^e^* 1-octene incorporation content, calculated by ^1^H NMR.

## Data Availability

Theoretical and experimental results are available from correspondence with the author.

## Supplementary Materials

The following supporting information can be downloaded at: https://www.mdpi.com/article/10.3390/polym16070902/s1, Figures S1–S14: NMR spectra of phenoxyimine [O^−^NO] ligands **L1**–**L7**; Figures S15–S28: NMR spectra of phenoxyimine [O^−^NO] titanium complexes **Ti1**–**Ti7**.
