# Supplementary material for: Thermal-Robust Phenoxyimine Titanium Catalysts Bearing Bulky Sidearms for High Temperature Ethylene Homo-/Co- Polymerizations
Source: Polymers (Basel). 2024 Mar 25;16(7):902. doi: 10.3390/polym16070902 (PMC11013879; doi:10.3390/polym16070902)
Supplement: Supplementary file 1 [file polymers-16-00902-s001.zip › polymers-2845306-supplementary.pdf]

Supporting Information

Contents

1. NMR spectra of phenoxyimine [O$^-$NO] ligands **L1** – **L7**.

2. NMR spectra of phenoxyimine [O$^-$NO] titanium complexes **Ti1**-**Ti7**.

1. NMR spectra of phenoxyimine [O⁻NO] ligands L1 – L7.

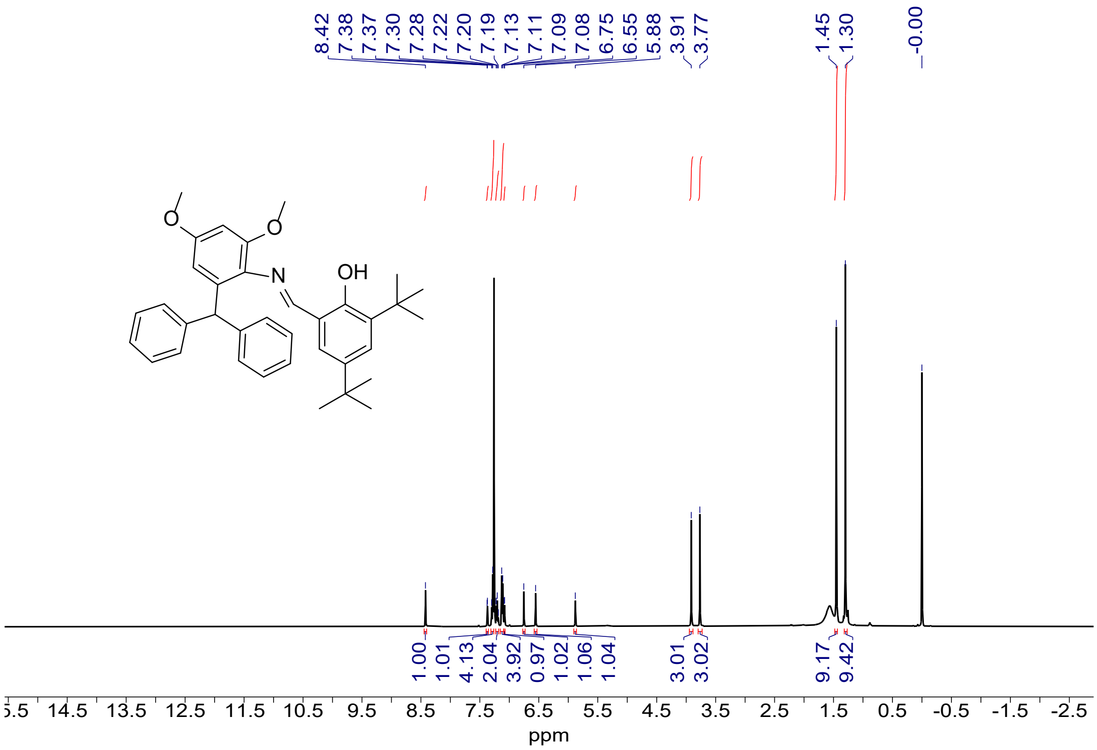

Figure S1. ^{1}H NMR spectrum (400 MHz, Chloroform-*d*, 298 K) of **L1.**

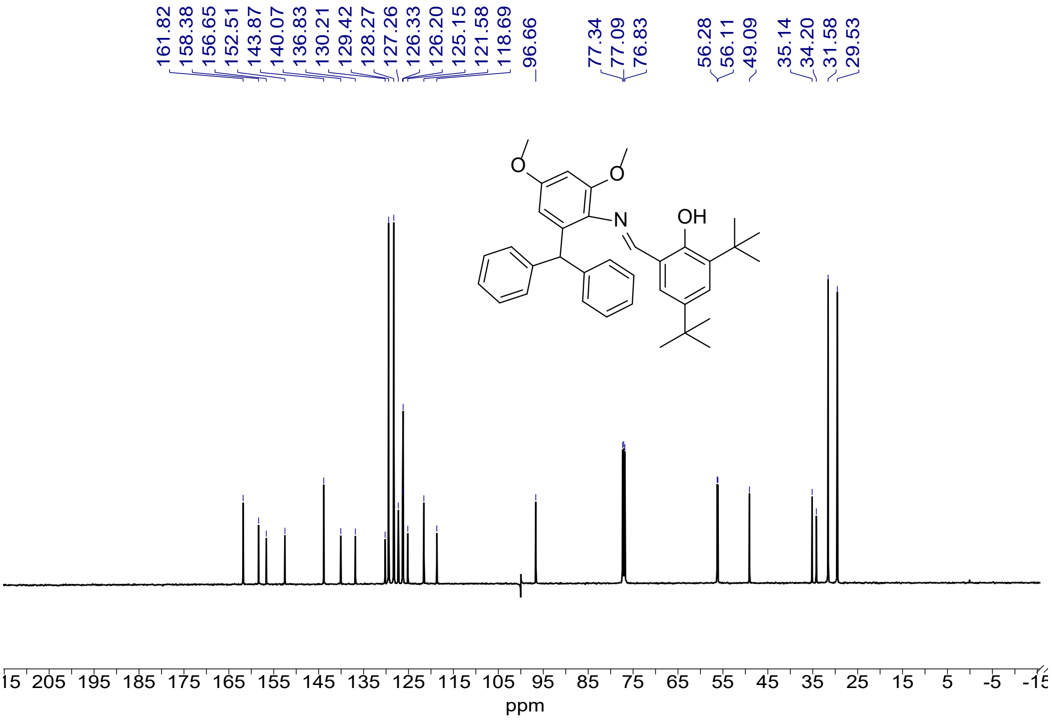

Figure S2. ^{13}C NMR spectrum (126 MHz, $CDCl_3$, 298 K) of **L1.**

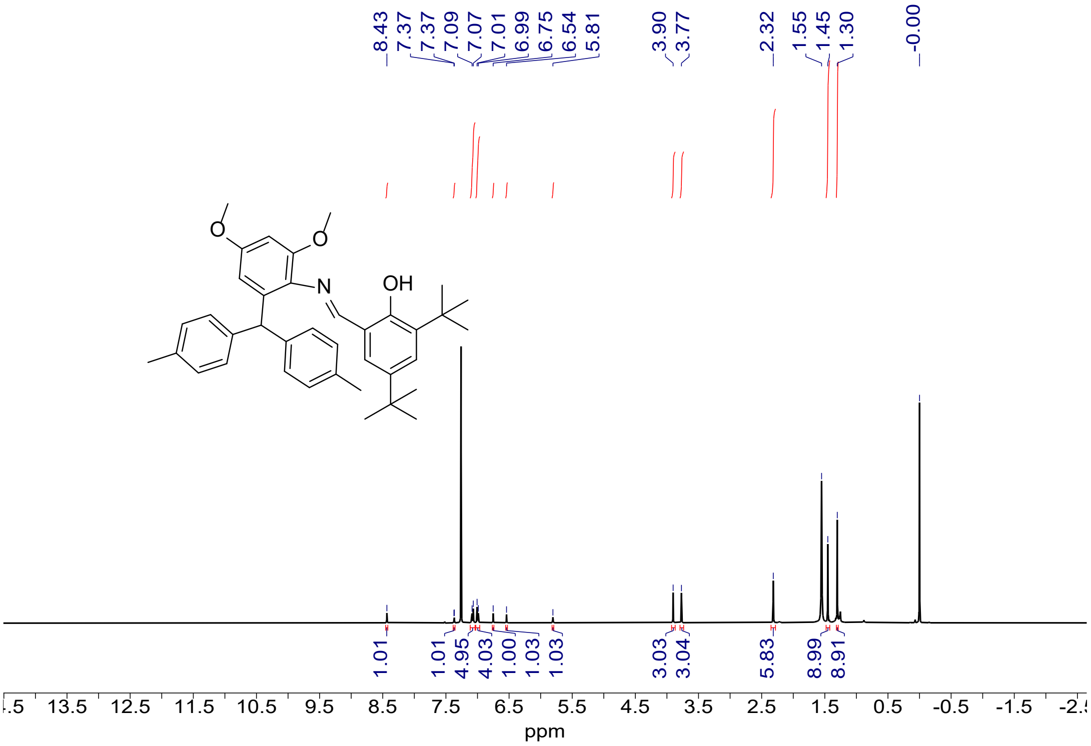

Figure S3. ^{1}H NMR spectrum (400 MHz, Chloroform-*d*, 298 K) of **L2.**

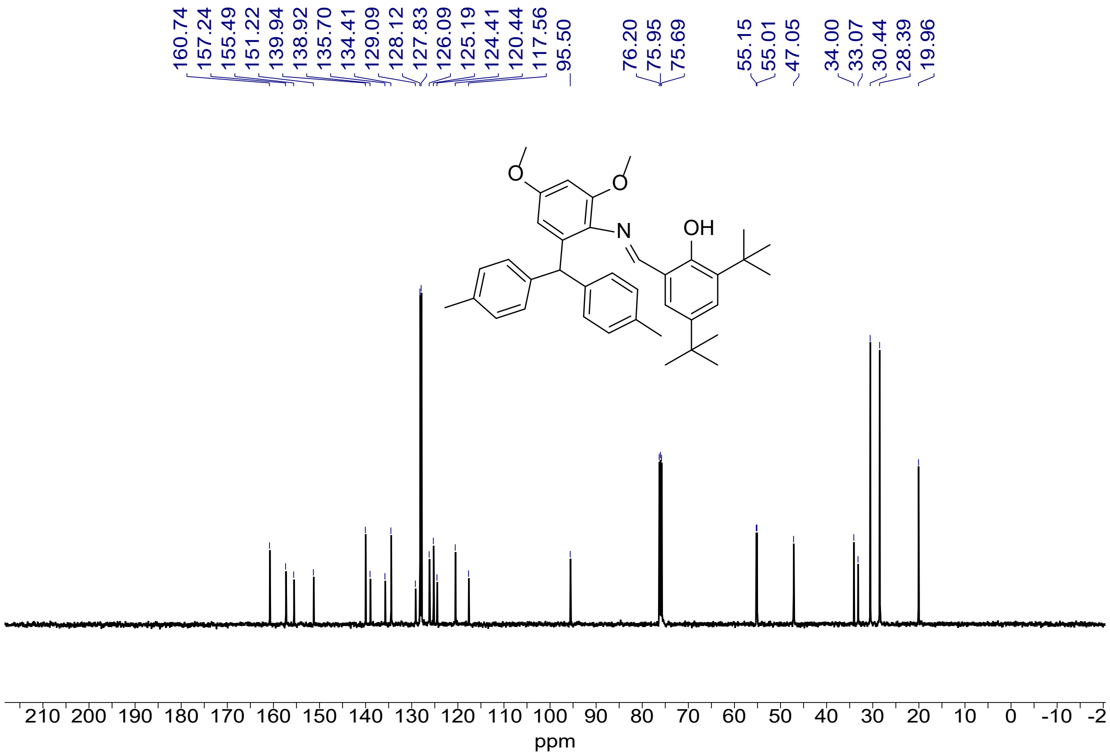

Figure S4. ^{13}C NMR spectrum (126 MHz, $CDCl_3$, 298 K) of **L2.**

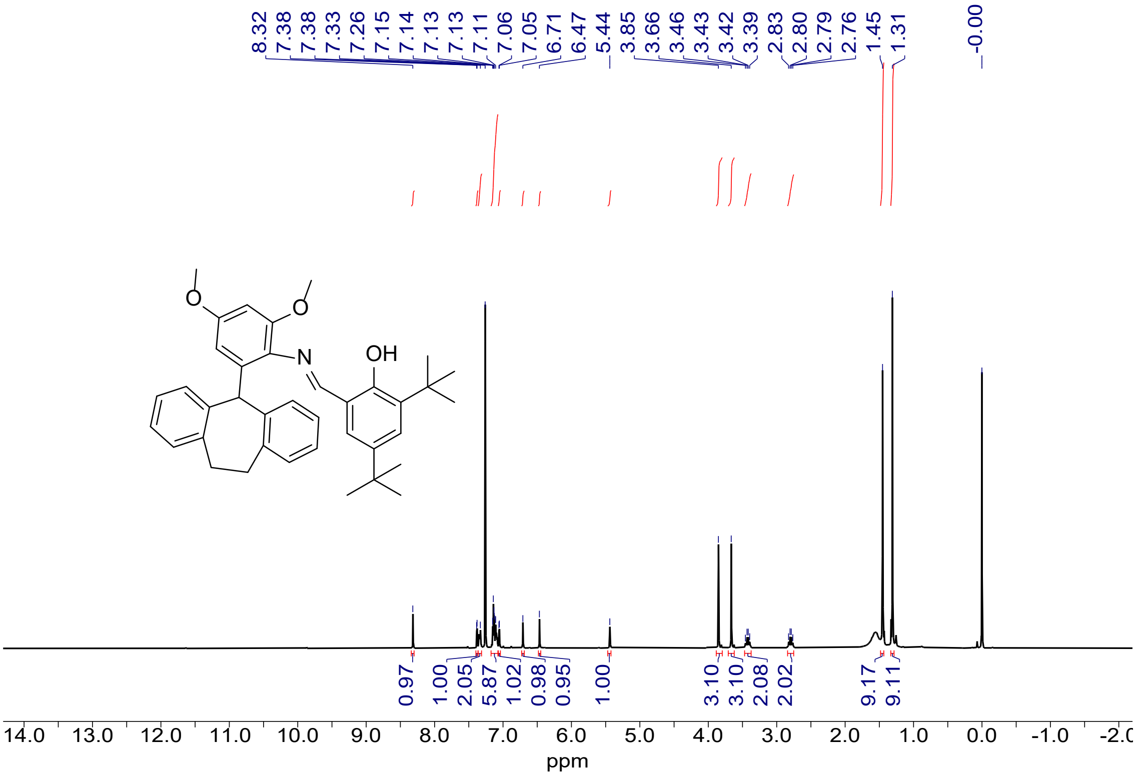

Figure S5. ^{1}H NMR spectrum (400 MHz, Chloroform-*d*, 298 K) of **L3.**

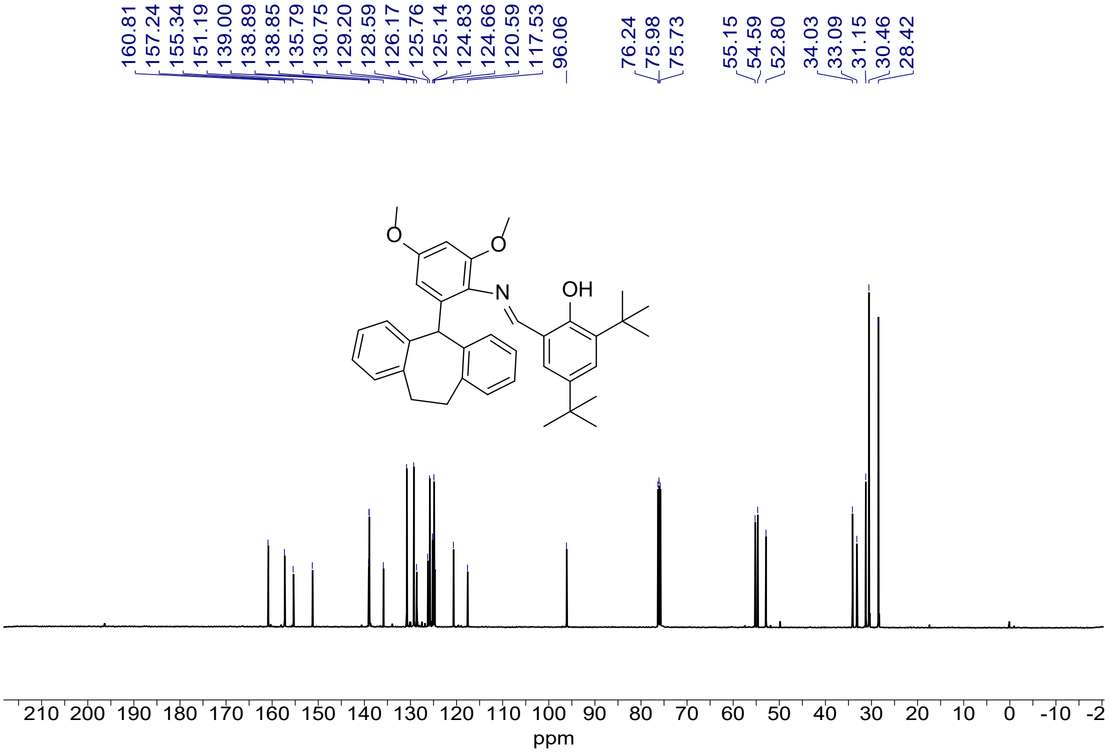

Figure S6. ^{13}C NMR spectrum (126 MHz, $CDCl_3$, 298 K) of **L3**

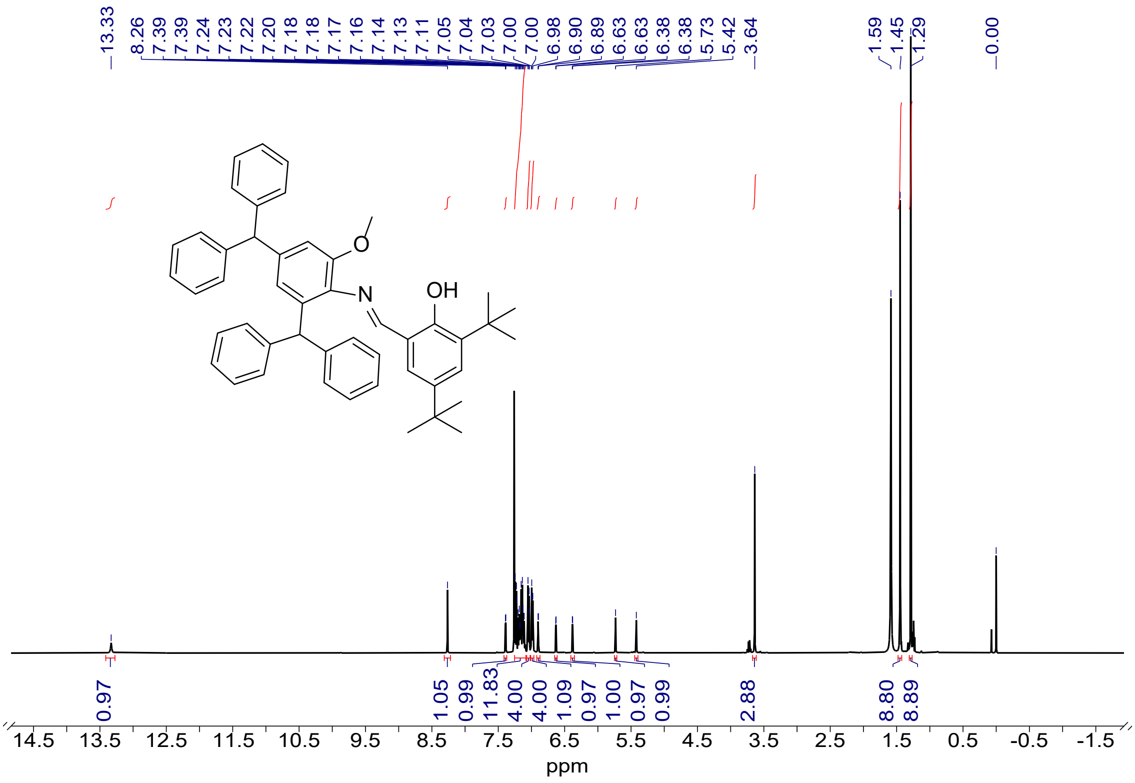

Figure S7. ^{1}H NMR spectrum (400 MHz, Chloroform-*d*, 298 K) of **L4.**

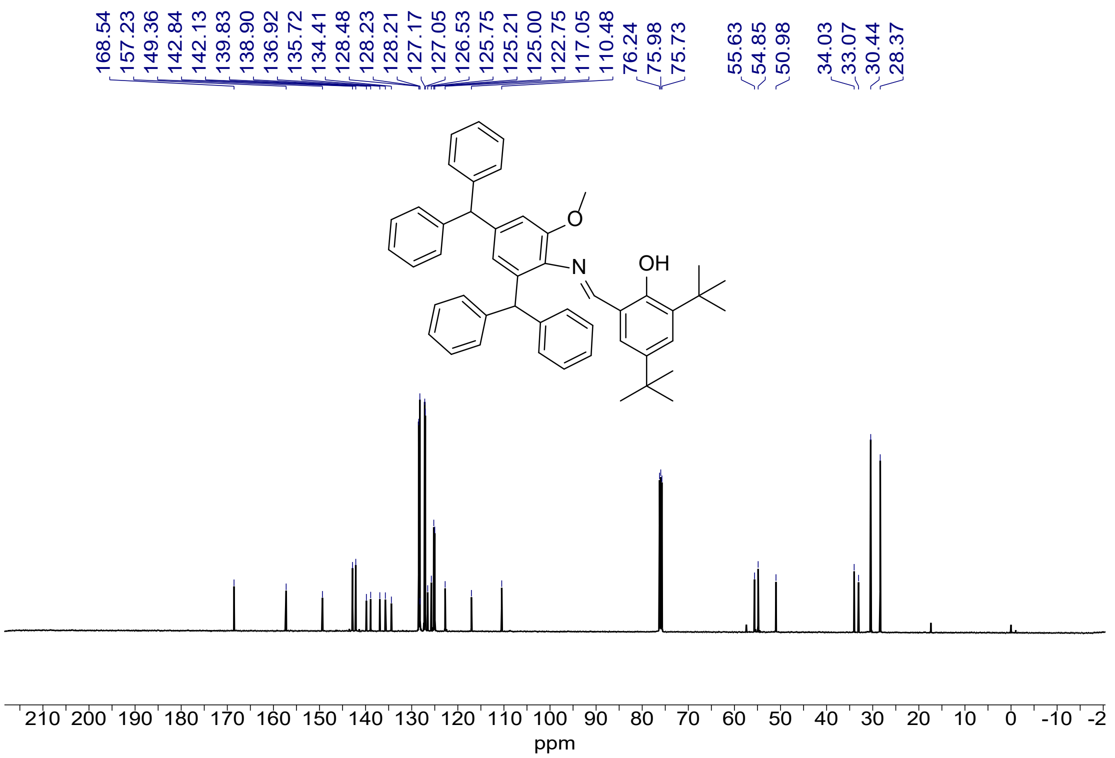

Figure S8. ^{13}C NMR spectrum (126 MHz, $CDCl_3$, 298 K) of **L4**

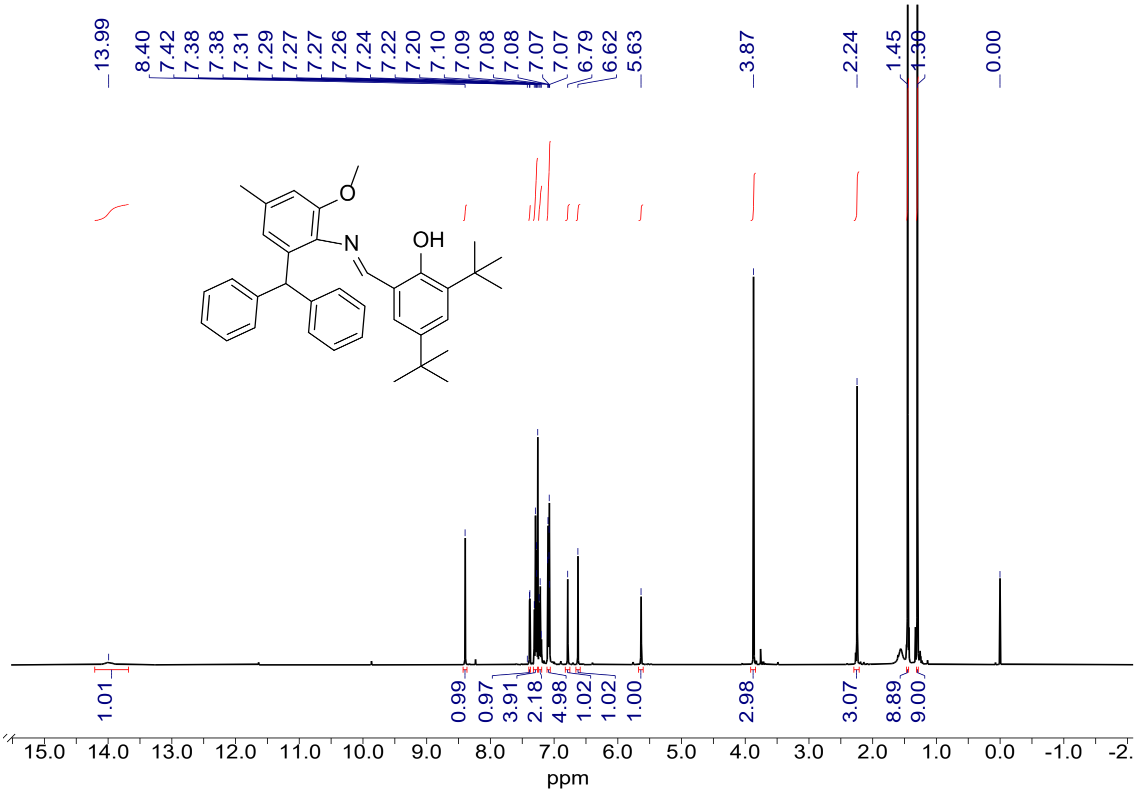

Figure S9. ^{1}H NMR spectrum (400 MHz, Chloroform-*d*, 298 K) of **L5.**

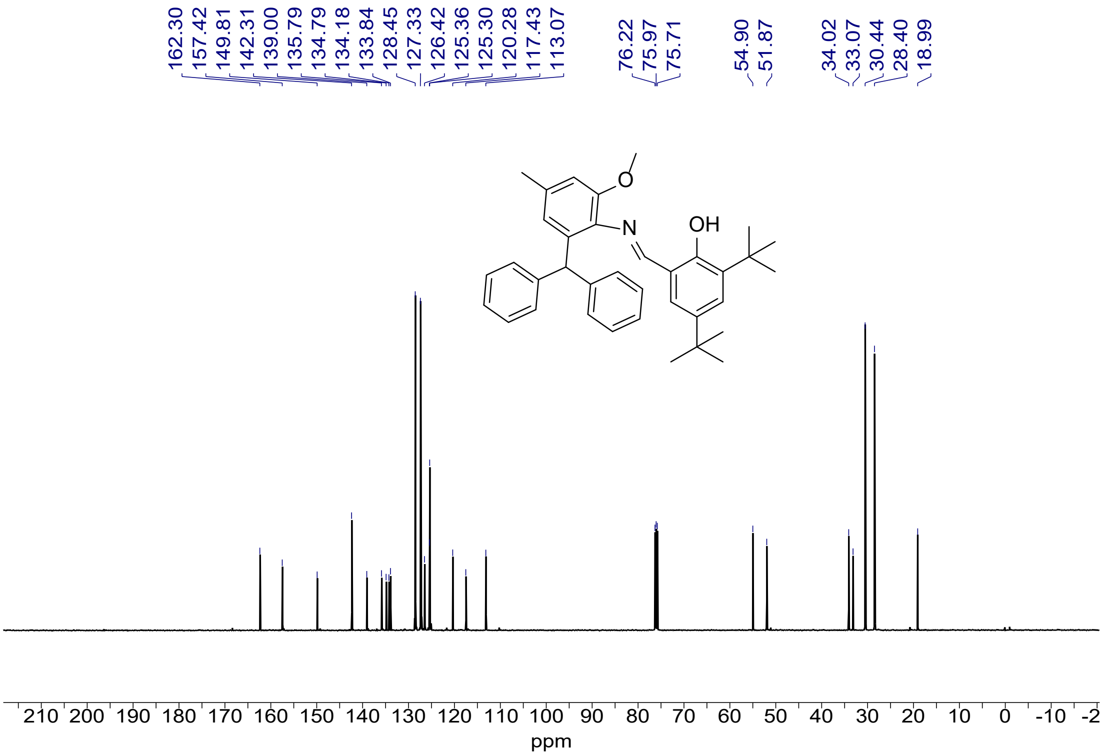

Figure S10. ^{13}C NMR spectrum (126 MHz, $CDCl_3$, 298 K) of **L5.**

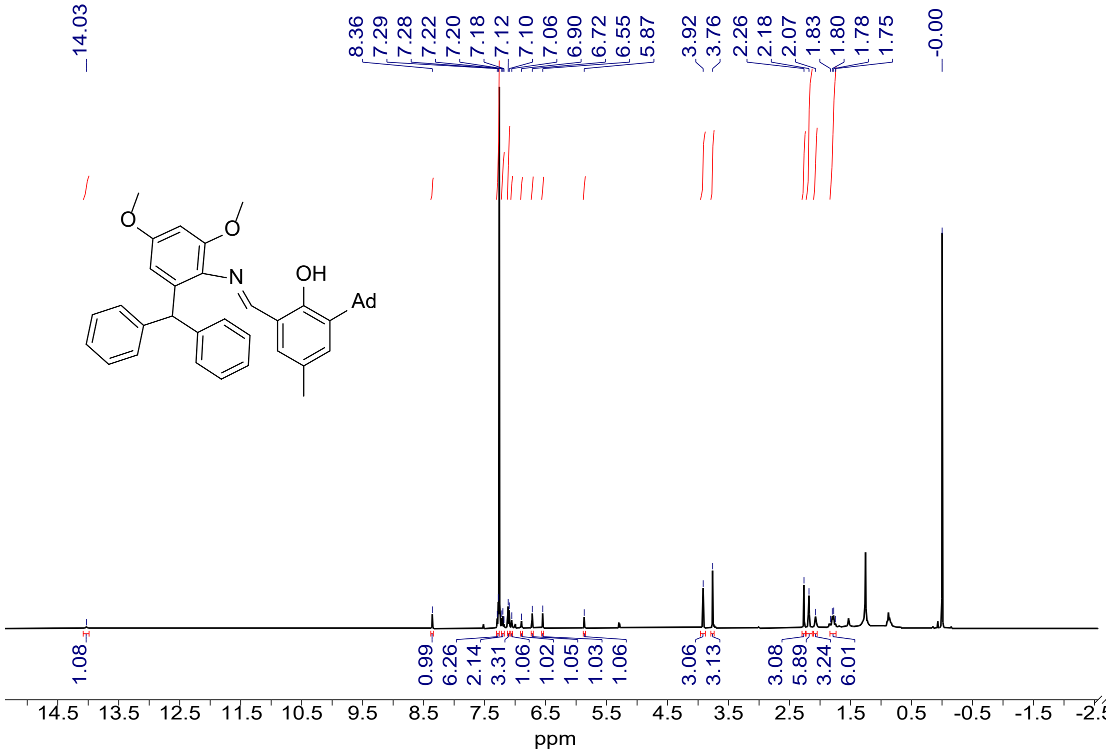

Figure S11. ^{1}H NMR spectrum (400 MHz, Chloroform-*d*, 298 K) of **L6.**

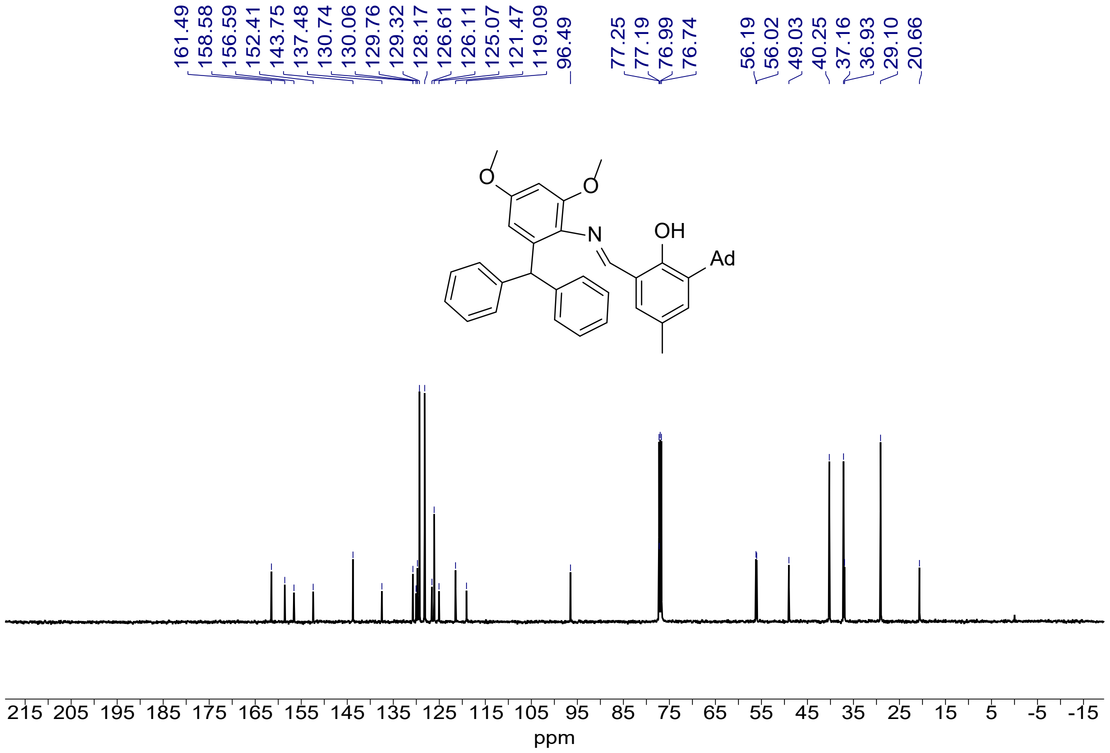

Figure S12. ^{13}C NMR spectrum (126 MHz, $CDCl_3$, 298 K) of **L6.**

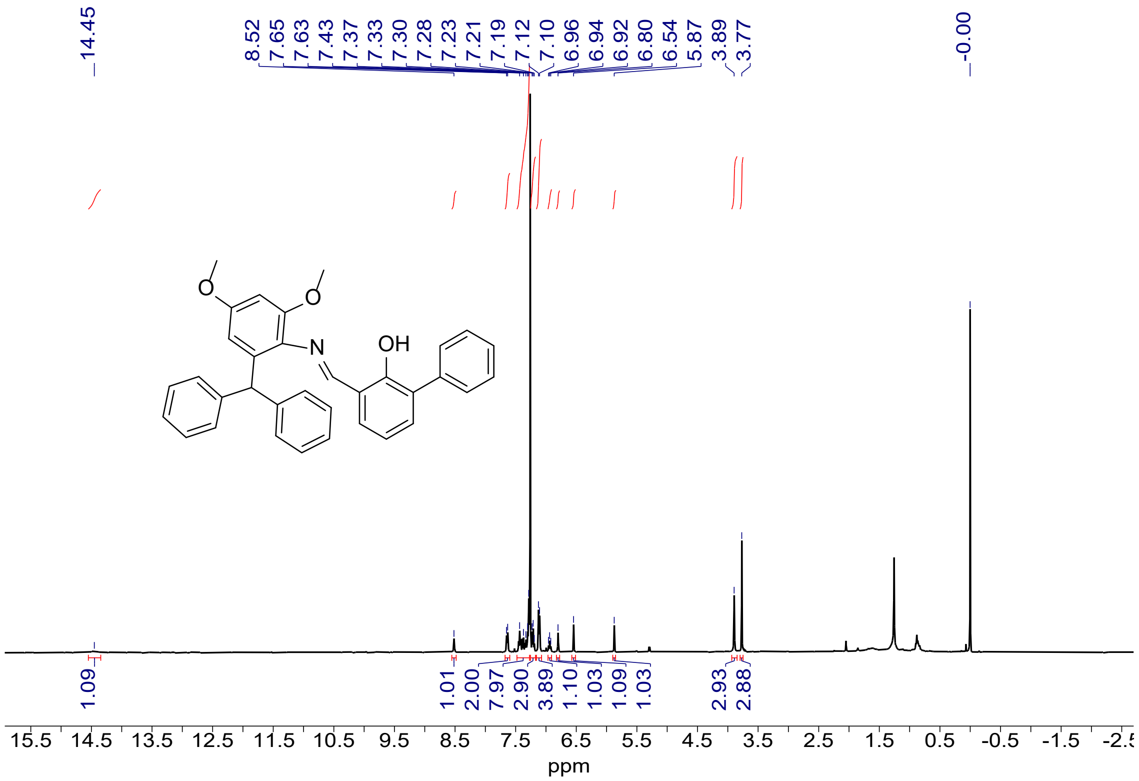

Figure S13. ^{1}H NMR spectrum (400 MHz, Chloroform-*d*, 298 K) of **L7.**

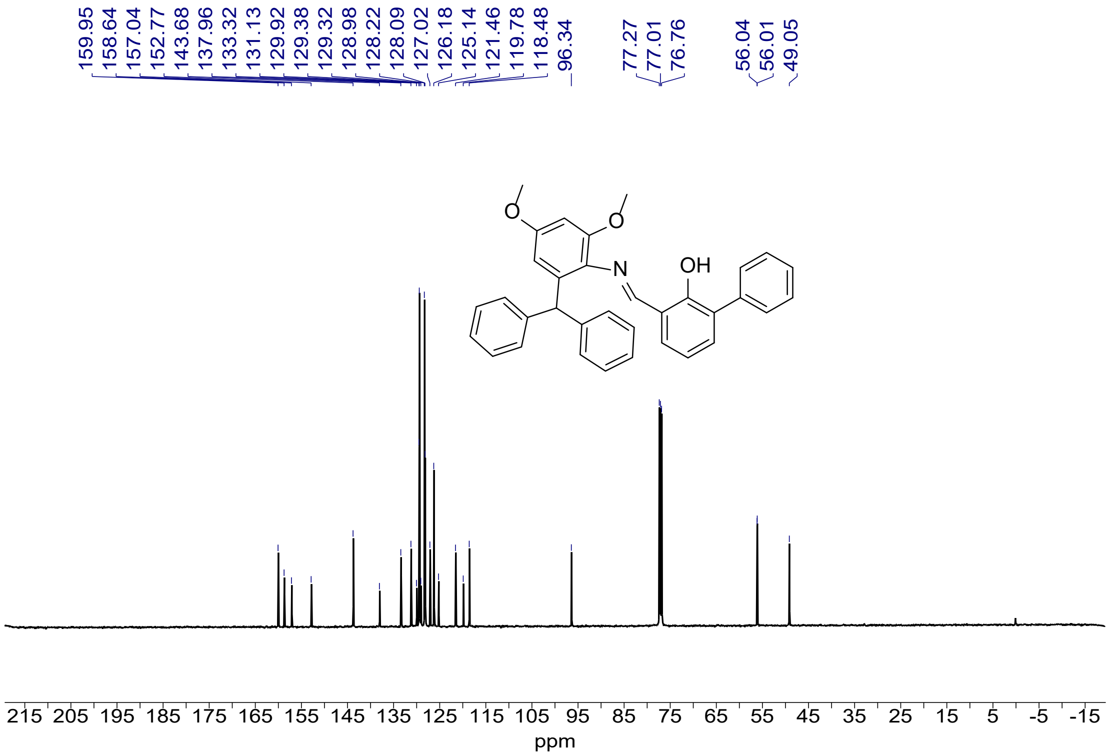

Figure S14. ^{13}C NMR spectrum (126 MHz, $CDCl_3$, 298 K) of **L7.**

2. NMR spectra of phenoxyimine [O-NO] titanium complexes Ti1-Ti7.

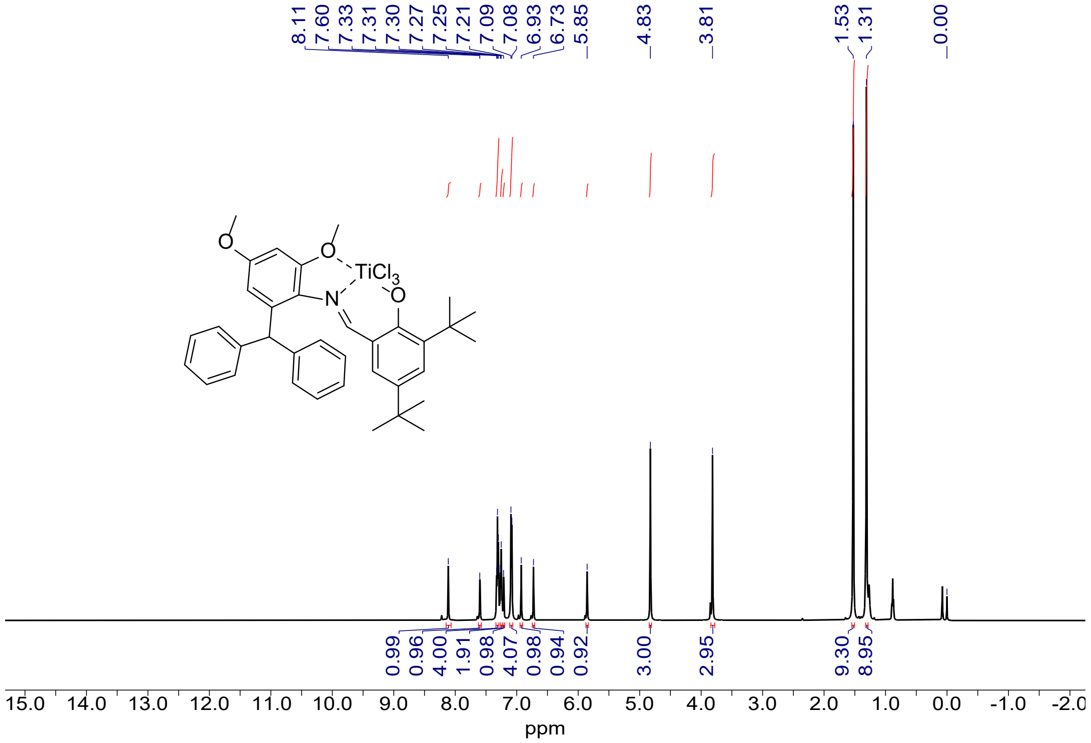

Figure S15. ^{1}H NMR spectrum (400 MHz, Chloroform-*d*, 298 K) of **Ti1.**

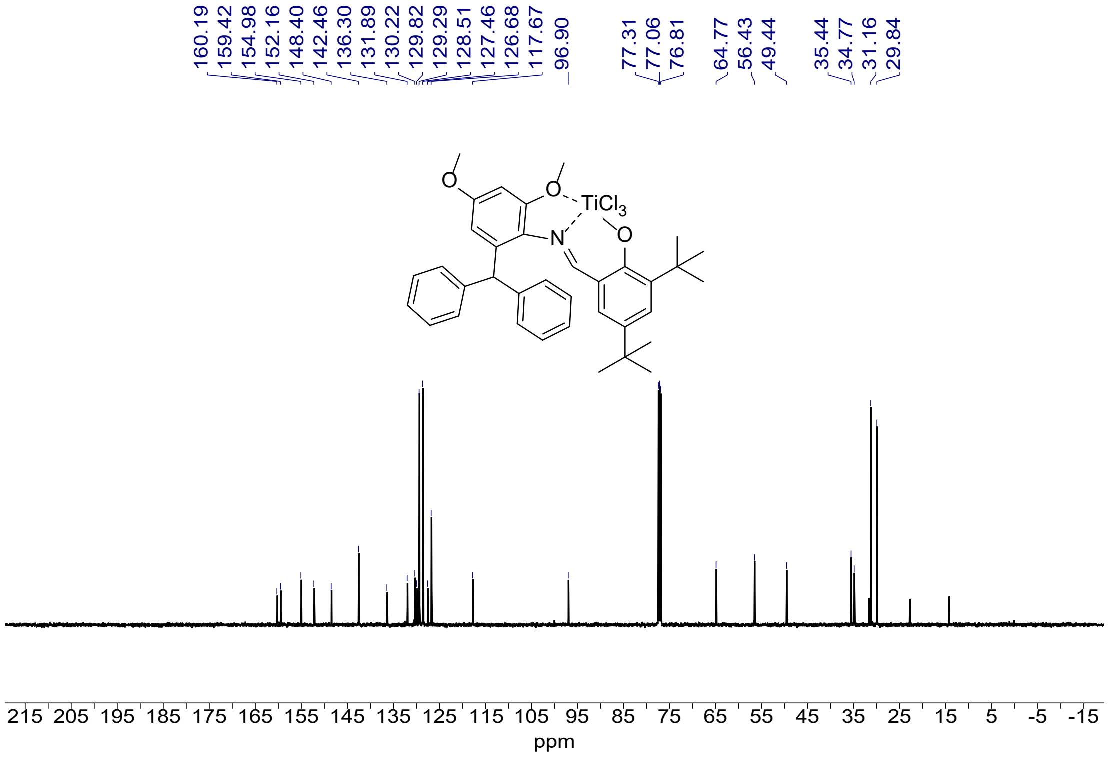

Figure S16. ^{13}C NMR spectrum (126 MHz, $CDCl_3$, 298 K) of **Ti1.**

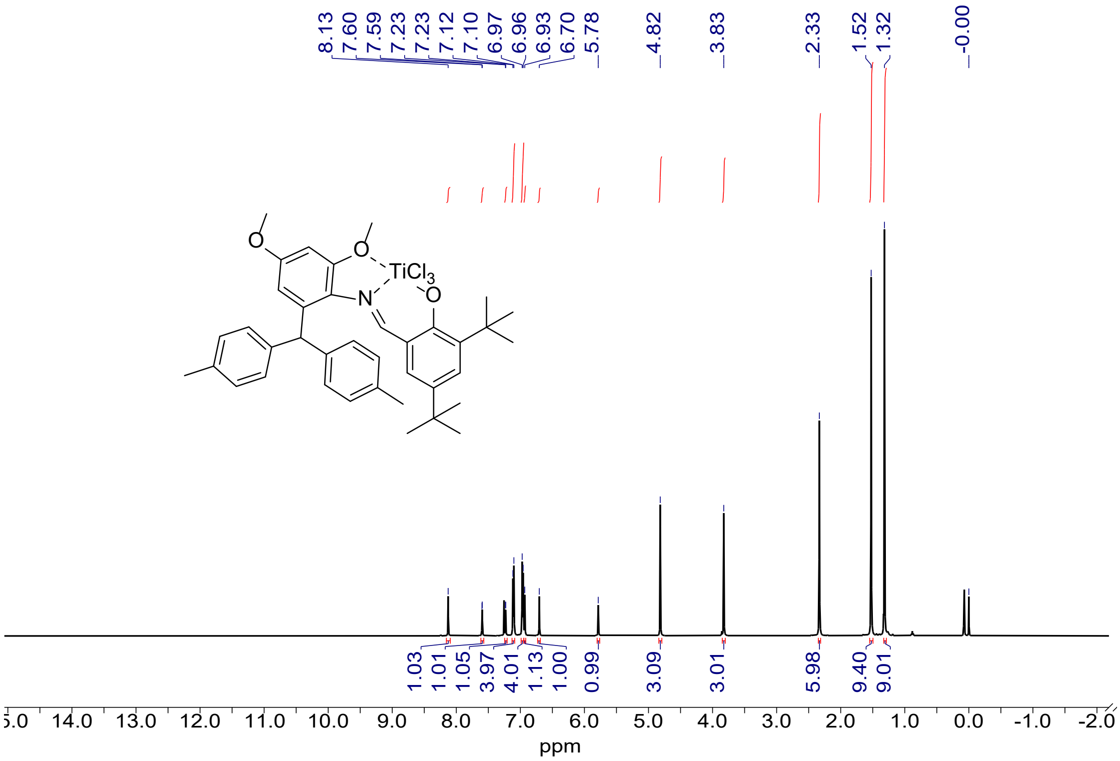

Figure S17. ^{1}H NMR spectrum (400 MHz, Chloroform-*d*, 298 K) of **Ti2.**

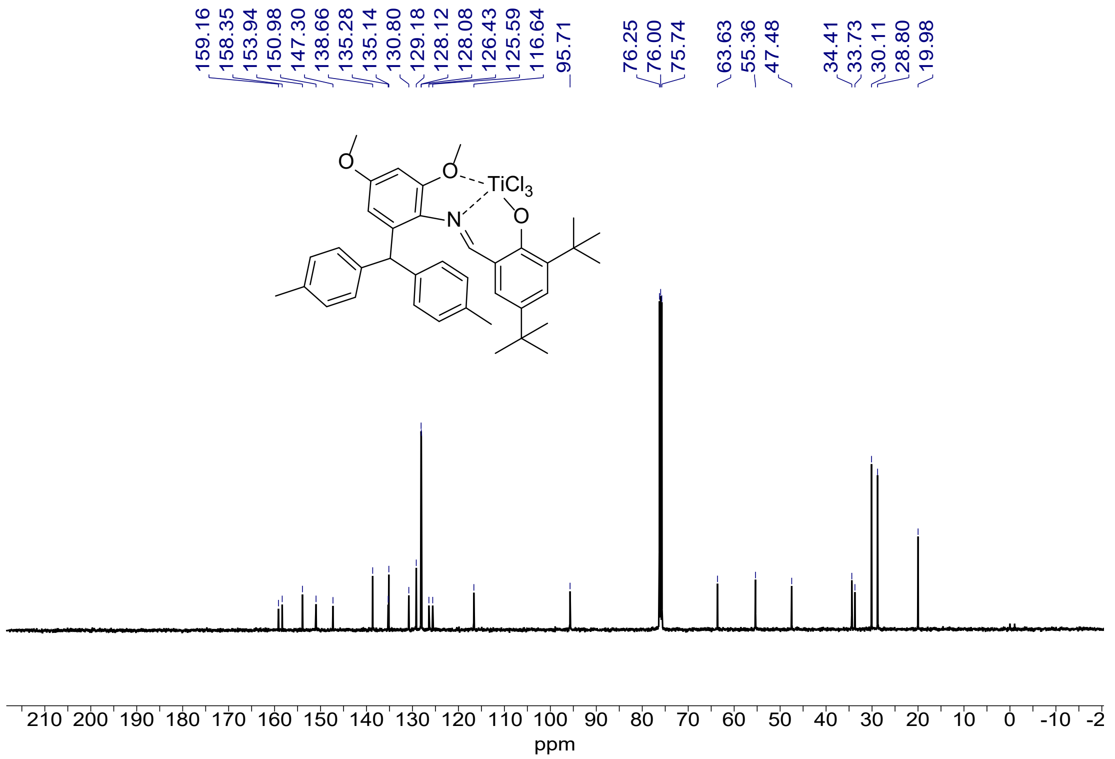

Figure S18. ^{13}C NMR spectrum (126 MHz, $CDCl_3$, 298 K) of **Ti2.**

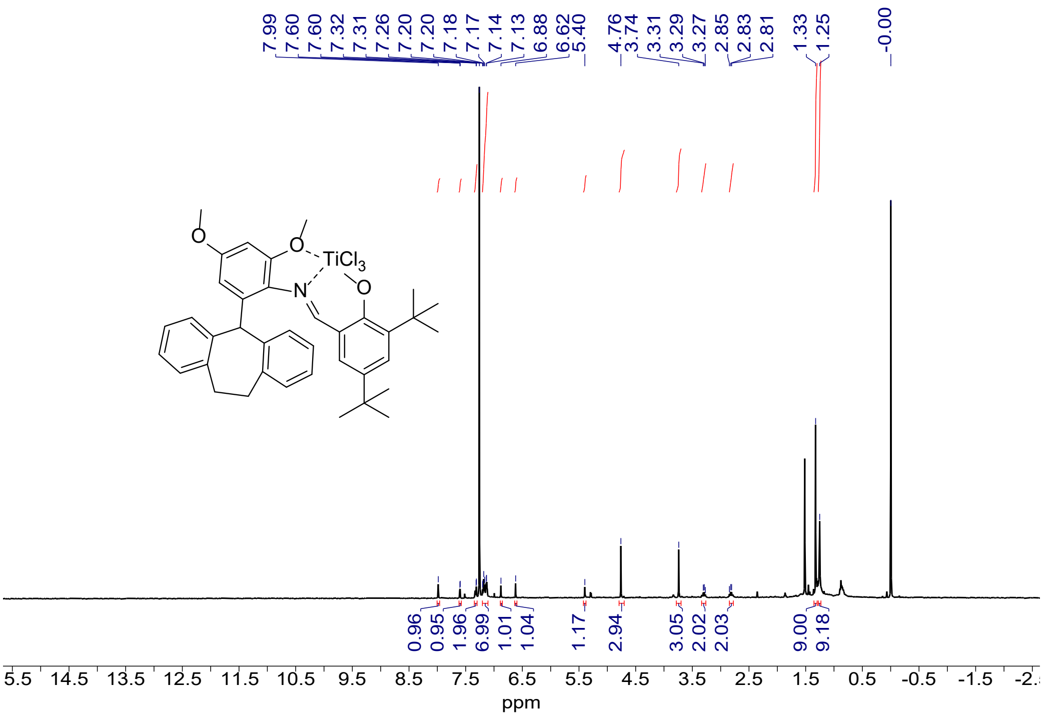

Figure S19. ^{1}H NMR spectrum (400 MHz, Chloroform-*d*, 298 K) of **Ti3.**

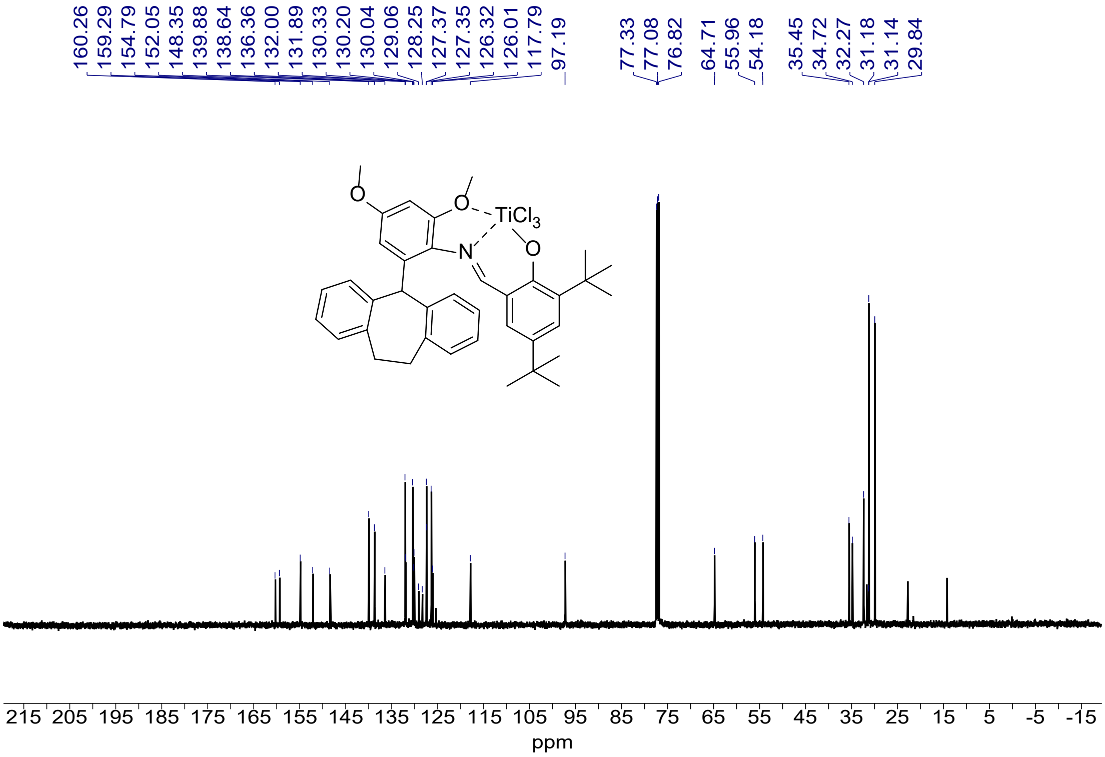

Figure S20. ^{13}C NMR spectrum (126 MHz, $CDCl_3$, 298 K) of **Ti3.**

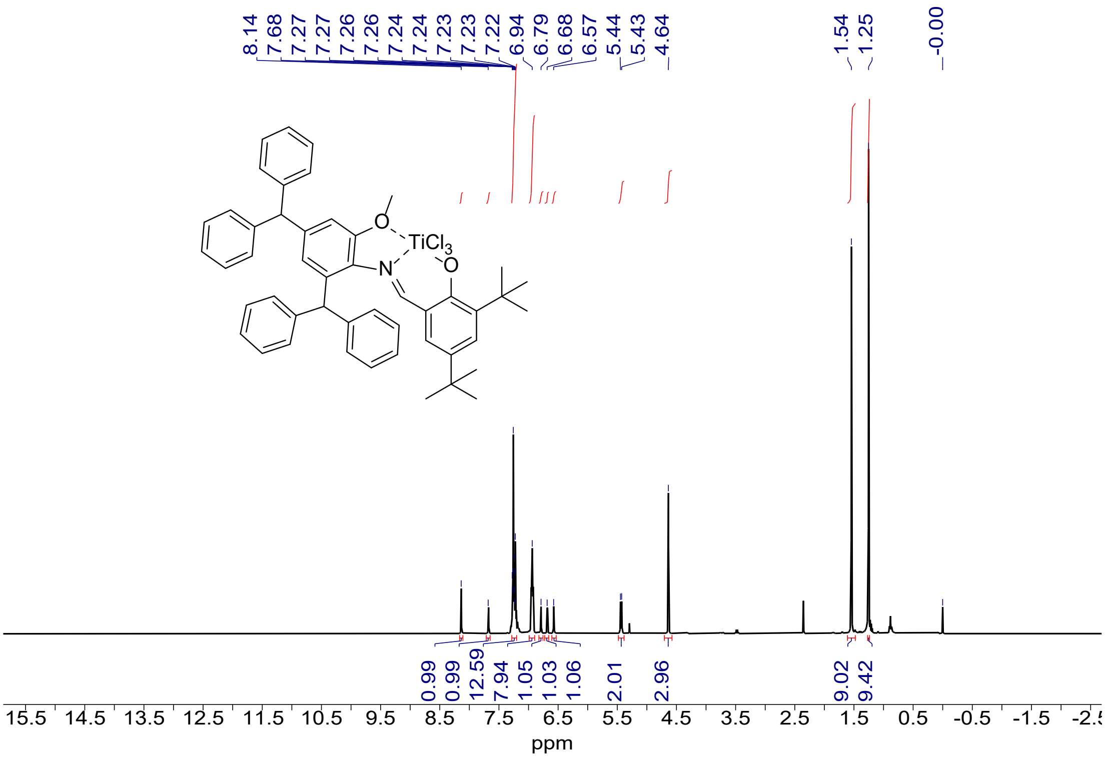

Figure S21. ^{1}H NMR spectrum (400 MHz, Chloroform-*d*, 298 K) of **Ti4.**

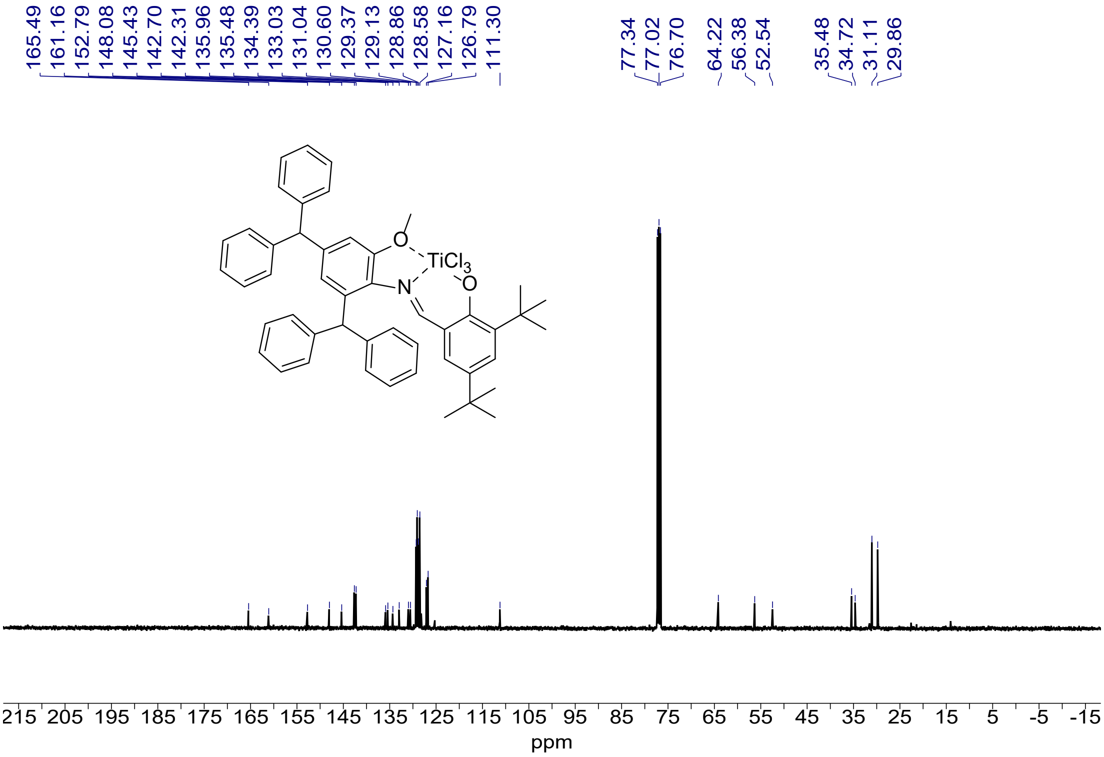

Figure S22. ^{13}C NMR spectrum (126 MHz, $CDCl_3$, 298 K) of **Ti4.**

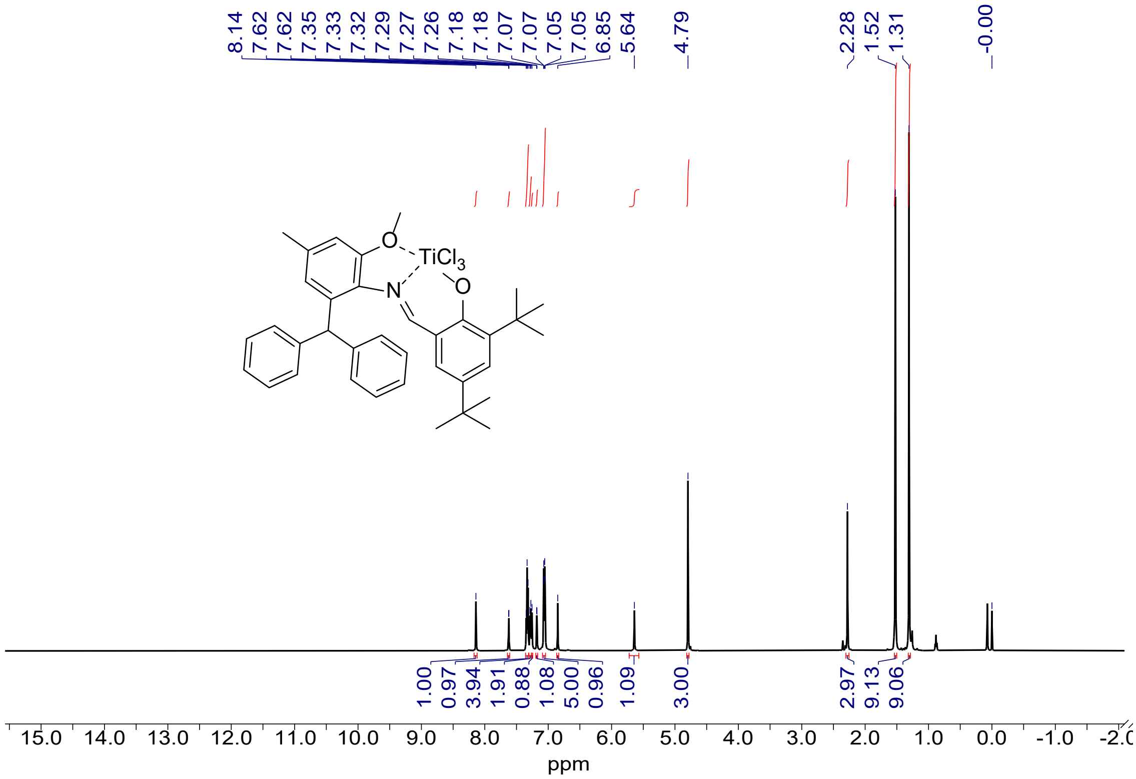

Figure S23. ^{1}H NMR spectrum (400 MHz, Chloroform-*d*, 298 K) of **Ti5.**

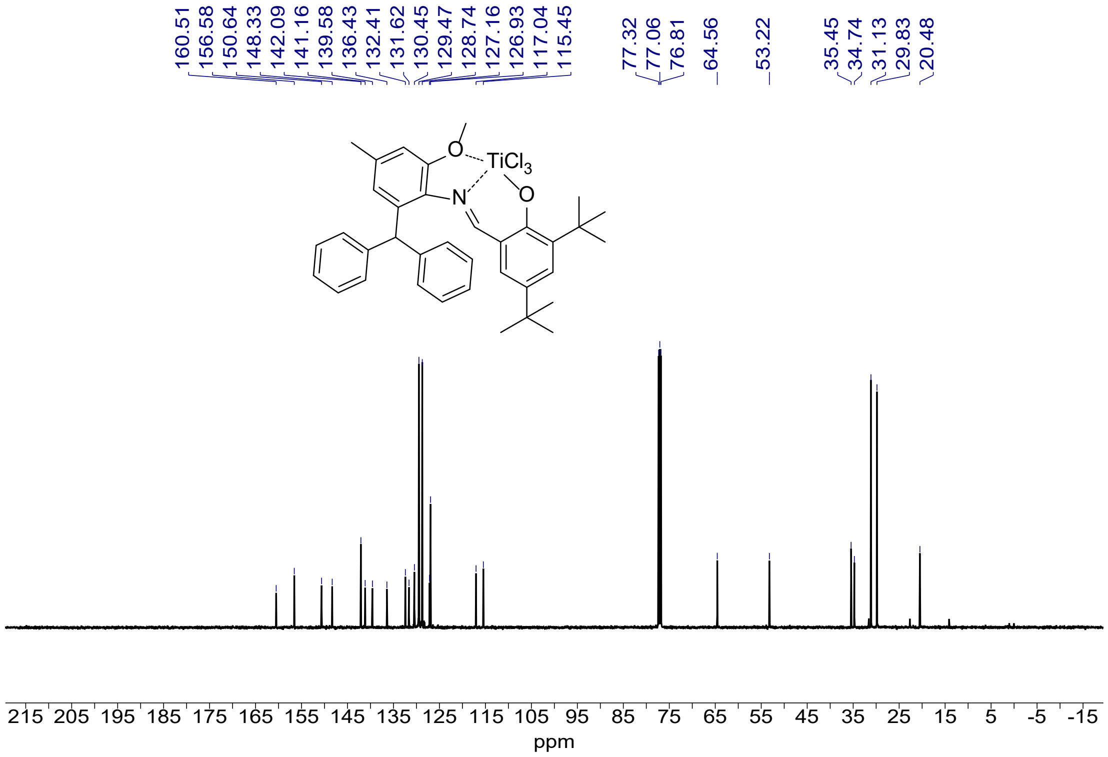

Figure S24. ^{13}C NMR spectrum (126 MHz, $CDCl_3$, 298 K) of of **Ti5.**

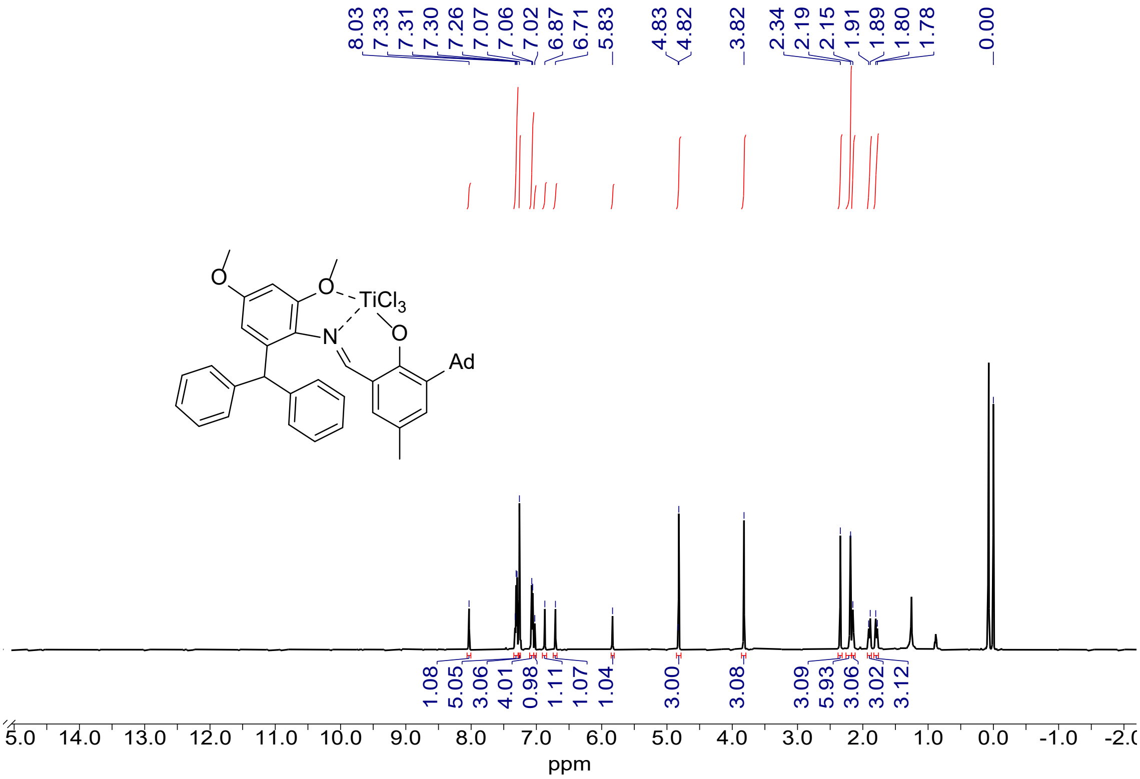

Figure S25. ^{1}H NMR spectrum (400 MHz, Chloroform-*d*, 298 K) of **Ti6.**

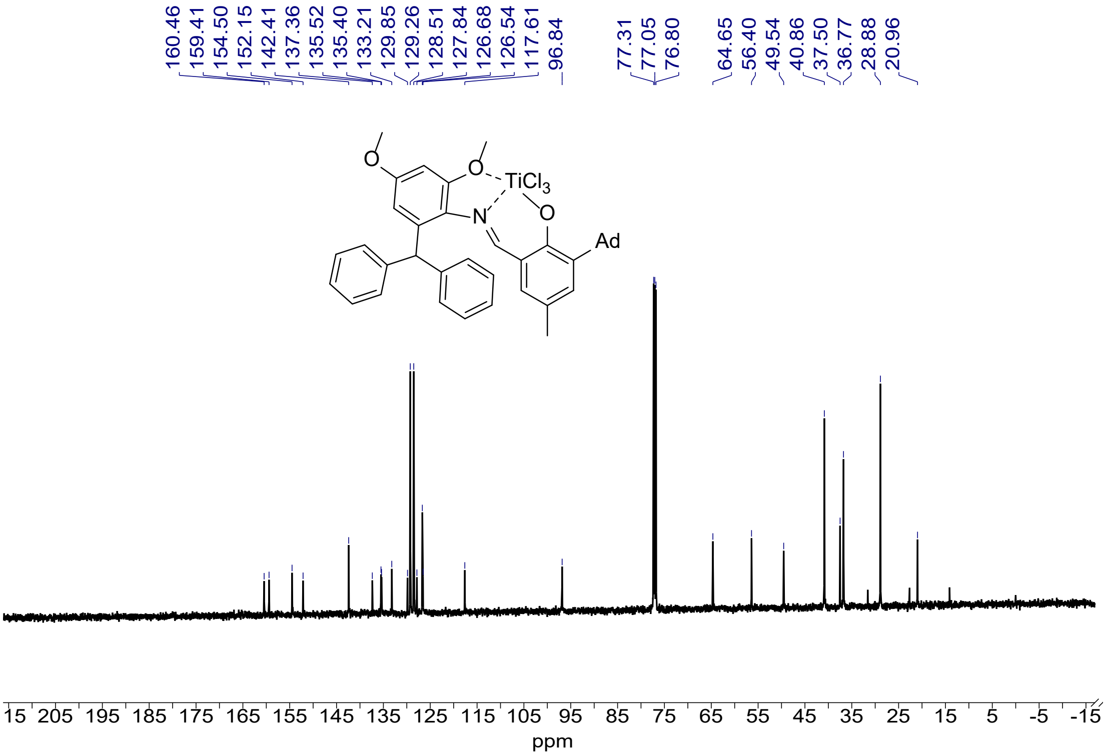

Figure S26. ^{13}C NMR spectrum (126 MHz, $CDCl_3$, 298 K) of **Ti6.**

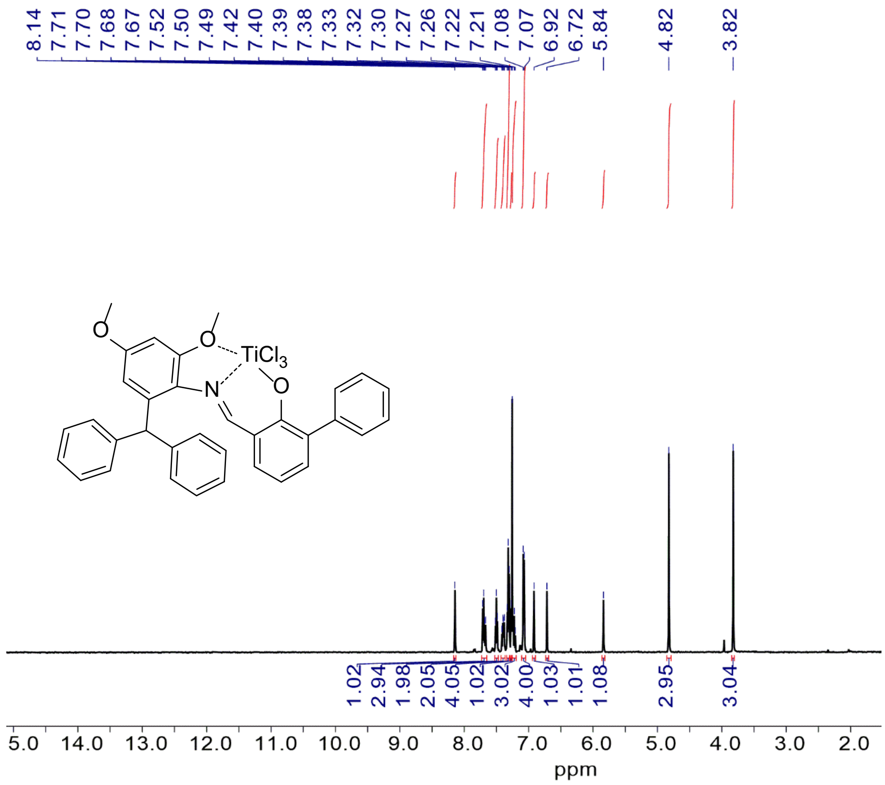

Figure S27. ^{1}H NMR spectrum (400 MHz, Chloroform-*d*, 298 K) of **Ti7.**

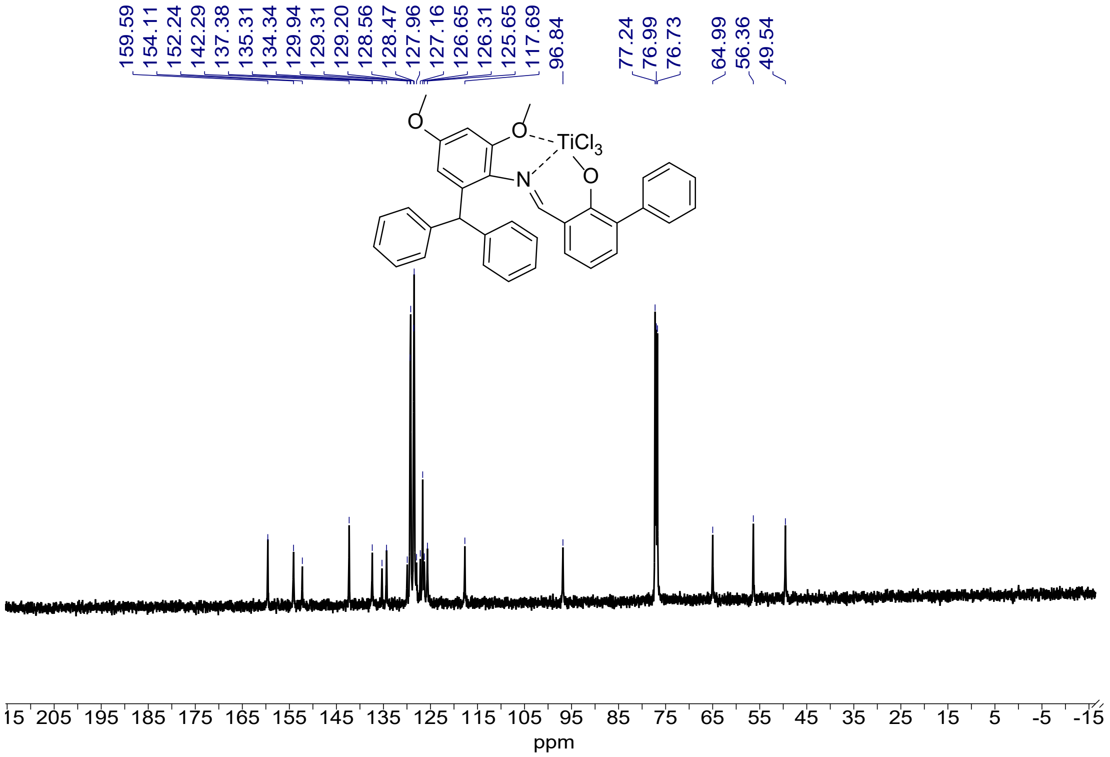

Figure S28. ^{13}C NMR spectrum (126 MHz, $CDCl_3$, 298 K) of **Ti7.**
